# Empirical estimates of regional carbon budgets imply reduced global soil heterotrophic respiration

**DOI:** 10.1093/nsr/nwaa145

**Published:** 2020-07-07

**Authors:** Philippe Ciais, Yitong Yao, Thomas Gasser, Alessandro Baccini, Yilong Wang, Ronny Lauerwald, Shushi Peng, Ana Bastos, Wei Li, Peter A Raymond, Josep G Canadell, Glen P Peters, Rob J Andres, Jinfeng Chang, Chao Yue, A Johannes Dolman, Vanessa Haverd, Jens Hartmann, Goulven Laruelle, Alexandra G Konings, Anthony W King, Yi Liu, Sebastiaan Luyssaert, Fabienne Maignan, Prabir K Patra, Anna Peregon, Pierre Regnier, Julia Pongratz, Benjamin Poulter, Anatoly Shvidenko, Riccardo Valentini, Rong Wang, Grégoire Broquet, Yi Yin, Jakob Zscheischler, Bertrand Guenet, Daniel S Goll, Ashley-P Ballantyne, Hui Yang, Chunjing Qiu, Dan Zhu

**Affiliations:** Laboratoire des Sciences du Climat et de l’Environnement, CEA-CNRS-UVSQ-UPSACLAY, Gif sur Yvette 91191, France; Sino-French Institute for Earth System Science, College of Urban and Environmental Sciences, Peking University, Beijing 100871, China; Laboratoire des Sciences du Climat et de l’Environnement, CEA-CNRS-UVSQ-UPSACLAY, Gif sur Yvette 91191, France; International Institute for Applied Systems Analysis (IIASA), Laxenburg A-2361, Austria; Woods Hole Research Center, Falmouth, MA 02540, USA; The Key Laboratory of Land Surface Pattern and Simulation, Institute of Geographical Sciences and Natural Resources Research, Chinese Academy of Sciences, Beijing 100871, China; Laboratoire des Sciences du Climat et de l’Environnement, CEA-CNRS-UVSQ-UPSACLAY, Gif sur Yvette 91191, France; Department Geoscience, Environment & Society, Université Libre de Bruxelles, Bruxelles 1050, Belgium; Sino-French Institute for Earth System Science, College of Urban and Environmental Sciences, Peking University, Beijing 100871, China; Department für Geographie, Ludwig-Maximilians-Universität München, München D-80333, Germany; Department of Earth System Science, Tsinghua University, Beijing 100084, China; Yale School of Forestry and Environmental Studies, Yale University, New Haven, CT 06511, USA; Global Carbon Project, CSIRO Oceans and Atmosphere, Canberra ACT 2601, Australia; CICERO Center for International Climate Research, Oslo 0349, Norway; Environmental Sciences Division and Climate Change Science Institute, Oak Ridge National Laboratory, Oak Ridge, TN 37831, USA; Laboratoire des Sciences du Climat et de l’Environnement, CEA-CNRS-UVSQ-UPSACLAY, Gif sur Yvette 91191, France; Laboratoire des Sciences du Climat et de l’Environnement, CEA-CNRS-UVSQ-UPSACLAY, Gif sur Yvette 91191, France; State Key Laboratory of Soil Erosion and Dryland Farming on the Loess Plateau, Northwest A&F University, Yangling 712100, China; Department of Earth Science, Vrije Universiteit Amsterdam, Amsterdam HV 1081, The Netherlands; CSIRO Oceans and Atmosphere, Canberra ACT 2601, Australia; Institute for Geology, CEN—Center for Earth System Research and Sustainability, University of Hamburg, Hamburg D-20146, Germany; Department Geoscience, Environment & Society, Université Libre de Bruxelles, Bruxelles 1050, Belgium; Department of Earth System Science, Stanford University, Stanford, CA 94305, USA; Environmental Sciences Division and Climate Change Science Institute, Oak Ridge National Laboratory, Oak Ridge, TN 37831, USA; School of Geographical Sciences, Nanjing University of Information Science and Technology, Nanjing 210044, China; Department of Ecological Sciences, Vrije Universiteit Amsterdam, Amsterdam HV 1081, The Netherlands; Laboratoire des Sciences du Climat et de l’Environnement, CEA-CNRS-UVSQ-UPSACLAY, Gif sur Yvette 91191, France; Research Institute for Global Change, JAMSTEC, Kanagawa 236-0001, Japan; Center for Environmental Remote Sensing, Chiba University, Chiba 263–8522, Japan; Laboratoire des Sciences du Climat et de l’Environnement, CEA-CNRS-UVSQ-UPSACLAY, Gif sur Yvette 91191, France; Institute of Soil Science and Agrochemistry, Siberian Branch of the Russian Academy of Sciences (SB RAS), Novosibirsk 630090, Russia; Tuva State University, Republic of Tuva, 667000, Russian; Department Geoscience, Environment & Society, Université Libre de Bruxelles, Bruxelles 1050, Belgium; Department Geoscience, Environment & Society, Université Libre de Bruxelles, Bruxelles 1050, Belgium; Max Planck Institute for Meteorology, Hamburg 20146, Germany; NASA Goddard Space Flight Center, Biospheric Sciences Lab., Greenbelt, MD 20771, USA; International Institute for Applied Systems Analysis (IIASA), Laxenburg A-2361, Austria; Department for Innovation in Biological, Agro-food and Forest systems (DIBAF), University of Tuscia, Viterbo 01100, Italy; RUDN University, Moscow 117198, Russia; Department of Environmental Science and Engineering, Shanghai Key Laboratory of Atmospheric Particle Pollution and Prevention, Institute of Atmospheric Sciences, Fudan University, Shanghai 200433, China; Laboratoire des Sciences du Climat et de l’Environnement, CEA-CNRS-UVSQ-UPSACLAY, Gif sur Yvette 91191, France; Division of Geological and Planetary Sciences, California Institute of Technology, Pasadena, CA 91125, USA; Climate and Environmental Physics and Oeschger Centre for Climate Change Research, University of Bern, Bern 3012, Switzerland; Laboratoire des Sciences du Climat et de l’Environnement, CEA-CNRS-UVSQ-UPSACLAY, Gif sur Yvette 91191, France; Laboratoire des Sciences du Climat et de l’Environnement, CEA-CNRS-UVSQ-UPSACLAY, Gif sur Yvette 91191, France; Department of Ecosystem and Conservation Science, University of Montana, Missoula, MT 59801, USA; Laboratoire des Sciences du Climat et de l’Environnement, CEA-CNRS-UVSQ-UPSACLAY, Gif sur Yvette 91191, France; Laboratoire des Sciences du Climat et de l’Environnement, CEA-CNRS-UVSQ-UPSACLAY, Gif sur Yvette 91191, France; Laboratoire des Sciences du Climat et de l’Environnement, CEA-CNRS-UVSQ-UPSACLAY, Gif sur Yvette 91191, France

**Keywords:** carbon budget, human appropriation of ecosystems, soil carbon

## Abstract

Resolving regional carbon budgets is critical for informing land-based mitigation policy. For nine regions covering nearly the whole globe, we collected inventory estimates of carbon-stock changes complemented by satellite estimates of biomass changes where inventory data are missing. The net land–atmospheric carbon exchange (NEE) was calculated by taking the sum of the carbon-stock change and lateral carbon fluxes from crop and wood trade, and riverine-carbon export to the ocean. Summing up NEE from all regions, we obtained a global ‘bottom-up’ NEE for net land anthropogenic CO_2_ uptake of –2.2 ± 0.6 PgC yr^−1^ consistent with the independent top-down NEE from the global atmospheric carbon budget during 2000–2009. This estimate is so far the most comprehensive global bottom-up carbon budget accounting, which set up an important milestone for global carbon-cycle studies. By decomposing NEE into component fluxes, we found that global soil heterotrophic respiration amounts to a source of CO_2_ of 39 PgC yr^−1^ with an interquartile of 33–46 PgC yr^−1^—a much smaller portion of net primary productivity than previously reported.

## REGIONAL NET LAND–ATMOSPHERE CARBON-EXCHANGE ESTIMATES FROM BOTTOM-UP INVENTORIES AND LATERAL CARBON FLUXES

Net ecosystem exchange (NEE) is defined as the land–atmosphere flux of carbon excluding fossil-fuel emissions [1,2]. Accurate and consistent observation-based estimates of NEE at regional scales are needed to understand the global land-carbon sink, to evaluate land-carbon models used for carbon-budget assessments and future climate projections and to define baselines for land-based mitigation efforts. Currently, land-carbon models show significant disagreement in their quantification of regional carbon fluxes [3,4]. A comprehensive global assessment of regional NEE from bottom-up approaches using land observations is still missing, which hampers an independent evaluation of the top-down global NEE deduced from the atmospheric CO_2_ growth rate [5]. In addition, understanding the components of regional NEE allows the turnover time of carbon lost to the atmosphere by soil heterotrophic respiration (SHR) and other processes and the response of land-carbon storage to increasing atmospheric CO_2_ (β) and warming (γ) to be better quantified [6].

We quantified NEE and its component fluxes for nine large land regions that cover nearly the entire land surface, using land-carbon-stock-change estimates reported from different inventories and observation-based data [7–16]. Special attention was brought to include uncertainties reported in each original publication and to use different data sources wherever available to account for uncertainty between data sets (Supplementary Table 1). NEE, the net carbon flux exchanged between each region and the atmosphere, excluding fossil-fuel emissions, is diagnosed from:

(1) \begin{equation*} {\rm{NEE}} = \Delta {\rm{C }} + {F_{\textit {rivers}}} + {F_{\textit {trade}}}, \end{equation*}

where ΔC is the net carbon-stock change in living and dead biomass, soil carbon, wood and crop products; *F_rivers_* is the flux of particulate and dissolved organic and inorganic carbon lost by each region to the ocean; and *F_trade_* is the net carbon flux from crop and wood products. *F_trade_* is a gain of carbon by a region if it imports more than it exports or a loss if otherwise (Supplementary Fig. 1). In Equation (1), the sign convention is that NEE is negative when a region removes CO_2_ from the atmosphere, ΔC is negative when carbon storage increases in that region, *F_rivers_* is always positive and *F_trade_* can be positive (net export) or negative (net import).

We estimated ΔC in each region based on publications from the REgional Carbon Cycle Assessment and Processes (RECCAP) project [7–14] completed by other data sources (Supplementary Table 1). The nine RECCAP regions cover the entire land surface except the Middle East, Ukraine, Belarus, Kazakhstan and New Zealand. These latter regions, given their surface area and/or their sparse vegetation, represent only a small fraction of global ΔC (Supplementary Table 1). For humid forests in tropical South America and Africa, ΔC of biomass was provided from long-term forest-plots data [8,12], but there were no such data for Southeast Asia and South Asia. To fill this gap, we used biomass ΔC from remote-sensing data combining Lidar and optical measurements [17]. This remote-sensing-based estimate of biomass ΔC is consistent with data from forest plots in South America and Africa, giving support to using it for Southeast Asia and South Asia (Supplementary Fig. 2). Further, unlike for other regions where regional data on soil-carbon-stock changes, albeit uncertain, were available, soil-carbon ΔC was ignored in the tropical regions where no data exist with adequate coverage. We estimated from global carbon-model output that ignoring soil carbon in our estimate of tropical ΔC could lead to an average underestimate of 26%, which is still within the range of uncertainties (Supplementary Text 1 and Table 2). To assess *F_trade_*, we used statistics of the wood and crop-products trade converted into carbon units [18]. To assess *F_rivers_*, we calculated the total export of fluvial carbon to the oceans using data from references [19] and [20] and subtracted the fraction of fluvial carbon that originates from weathered carbonate minerals, which represents a transfer from the lithosphere to the ocean that does not involve a contemporary atmospheric C flux (see ‘Methods’ section). Error propagation was applied to each term of Equation (1) to quantify regional NEE uncertainties.

## A NEW GLOBAL ESTIMATION OF LAND–ATMOSPHERE NET CARBON EXCHANGE

**Figure 1.**
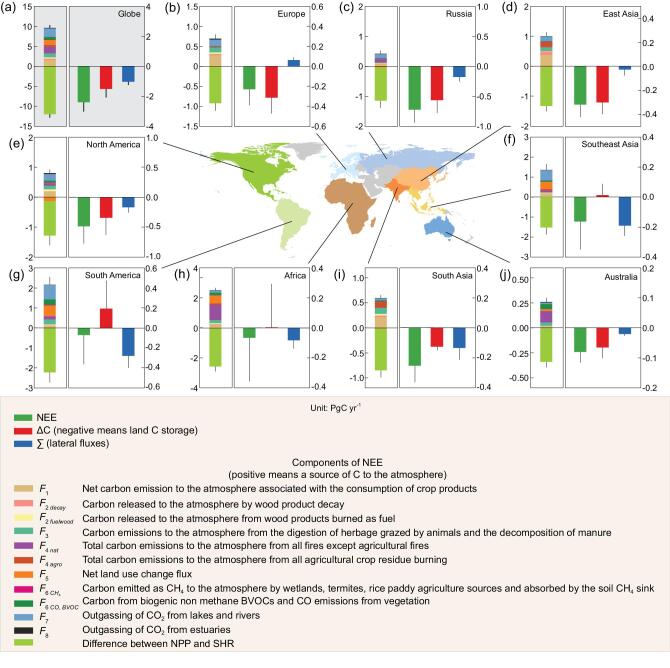
(a-j) Carbon-storage change from inventories (ΔC in red) and lateral fluxes from trade- and riverine-carbon export to the ocean for different regions of the globe (in blue) for the 2000s (Supplementary Fig. 1). The resulting bottom-up NEE from the sum of ΔC and lateral fluxes is given in green. An atmospheric convention is used, so that ΔC < 0 denotes an increase in land-carbon stocks and NEE < 0 is also a net uptake of atmospheric CO_2_. The upper stacked bars on the left show the NEE subcomponents that are sources of C to the atmosphere, excluding soil heterotrophic respiration, and the green bar is the resulting imbalance between the net primary productivity (NPP) and soil heterotrophic respiration, a negative value indicating that the soil heterotrophic respiration is smaller than NPP.

We obtained a bottom-up anthropogenic NEE of –2.2 ± 0.6 PgC yr^−1^, totally independently of the value derived from the global CO_2_ budget [5] of –2.4 ± 0.7 PgC yr^−1^ but remarkably consistent with it. Our bottom-up estimate is calculated from the sum of all the regional NEE values (Supplementary Table 1) plus NEE from regions not covered by the RECCAP publications being a sink of –0.4 PgC yr^−1^ (Supplementary Text 2 and Table 3). This gives a net uptake of atmospheric CO_2_ equal to –2.8 ± 0.7 PgC yr^−1^ for the period 2000–2009 (Fig. 1a). This estimate includes the sum of the anthropogenic sink linked to the human-induced perturbation of the global carbon cycle and the background natural CO_2_ sink associated with the fact that there is always a fraction of atmospheric CO_2_ absorbed on land by the vegetation, which is leached to rivers and transferred to the ocean [21]. In contrast, the NEE calculated from the global CO_2_ budget [5] only quantifies the anthropogenic land-CO_2_ uptake. We thus subtracted from our bottom-up NEE value the background sink from reference [21] to derive our bottom-up anthropogenic NEE estimate.

The contributions of each region to the global bottom-up NEE differ in magnitude. Tropical regions are small sinks of atmospheric CO_2_ and have NEE values of –0.25 ± 0.11 PgC yr^−1^ in South Asia, –0.17 ± 0.19 PgC yr^−1^ in Southeast Asia, –0.07 ± 0.29 PgC yr^−1^ in South America and –0.06 ± 0.29 PgC yr^−1^ in Africa, summing up to a small tropical land-CO_2_ sink of –0.55 ± 0.46 PgC yr^−1^. Northern-hemisphere regions tend to have larger CO_2_ sinks: –0.23 ± 0.16 PgC yr^−1^ in Europe, –0.32 ± 0.10 PgC yr^−1^ in East Asia, –0.49 ± 0.3 PgC yr^−1^ in North America and –0.73 ± 0.22 PgC yr^−1^ in Russia. The NEE from these northern regions amounts to –1.8 ± 0.4 PgC y^−1^, which is within the range of independent estimates given by atmospheric inversions [5].

## WHY LAND–ATMOSPHERE NET CARBON EXCHANGE DIFFERS FROM CARBON-STOCK CHANGES

Regional differences between NEE and ΔC are displayed in Fig. 1. In Russia, North America, South Asia and Australia, NEE is a larger CO_2_ sink than the increase in carbon stocks (ΔC) because a fraction of CO_2_ fixed from the atmosphere is exported by trade and rivers. In Europe, however, NEE is a smaller CO_2_ sink than ΔC because trade imports exceed riverine-carbon exports, given the fact that imported products are oxidized by humans and animals, releasing CO_2_ into the atmosphere in these two regions. In Africa, Southeast Asia and South America, inventories show a reduction in carbon stocks (positive ΔC values in Fig. 1) because of deforestation, but NEE is still a small net sink of atmospheric CO_2_ from the atmosphere, due to strong lateral exports from rivers and trade. These regional differences between NEE and ΔC clearly demonstrate that the results of top-down atmospheric inversions estimating NEE are not comparable to bottom-up carbon-stock change ΔC from inventories at the regional scale. We thus recommend that stock-change-based regional carbon budgets should be corrected for lateral fluxes to be properly compared with inversion results [11]. At the scale of smaller regions that exchange a lot of carbon by trade circuits, such as crop-production basins, plantation areas exporting carbon products or populated areas importing and consuming those products, differences between inversions NEE and ΔC are expected to be even larger in relative value.

## DEDUCING SHR CO_2_ EMISSIONS FROM NET ECOSYSTEM CARBON EXCHANGE

SHR is one of the largest and arguably the most uncertain flux of the global land-carbon cycle. Here, we combined our new bottom-up NEE estimates with other component fluxes exchanged with the atmosphere to infer SHR (‘Methods’ section and Supplementary Fig. 3). Regional values of net primary productivity (NPP) were taken from three satellite-based products [22–24] and a global land-carbon-cycle data-assimilation system [25]. These products based on different approaches and different satellite sensors give a global NPP of –50 PgC yr^−1^ with an interquartile range (IQR) of –57 to –44 PgC yr^−1^ over all RECCAP regions (Monte-Carlo standard deviation across the four NPP estimates and the uncertainty of each estimate; see ‘Methods’ section). Those four different NPP estimates are also consistent with each other for each individual region, within their respective uncertainties (Supplementary Fig. 4). Once fixed by NPP, carbon turns over in ecosystem pools and is returned back to the atmosphere mainly by SHR, but also by land-use-change emissions; fires; livestock grazing and the harvest wood and crop products subsequently oxidized by humans and animals; outgassing of carbon by lakes, rivers, and estuaries; and biogenic emissions of reduced-carbon compounds including methane and biogenic volatile organic compounds (Supplementary Fig. 3). All these gross fluxes and their uncertainties were estimated for each region using observational data sets, considering wherever possible different independent estimates for consistency checking (‘Methods’ section and Supplementary Table 1). Then, SHR, which is the largest single flux of CO_2_ lost by the land to the atmosphere, was calculated for each region as a residual, namely as the difference between NEE, NPP and all other non-SHR carbon exchanges. Uncertainties of SHR were obtained using a Monte-Carlo approach, sampling different estimates and their individual uncertainties (Supplementary Table 1).

**Figure 2.**
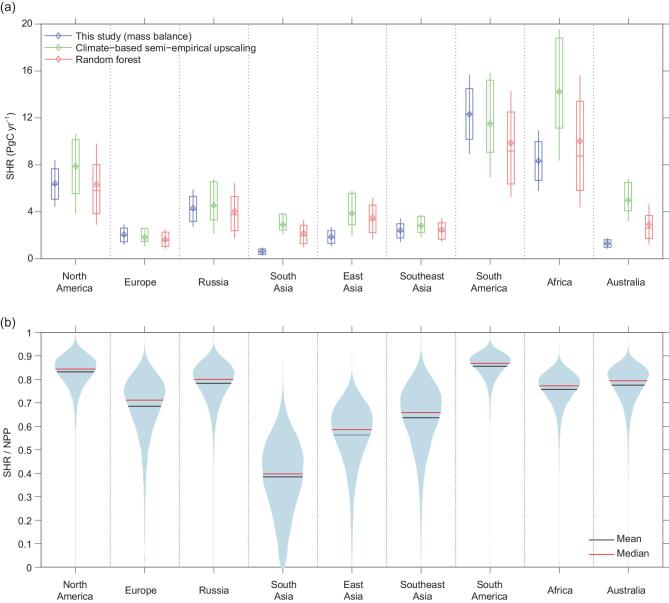
(a) Regional estimates of soil heterotrophic respiration from this study in blue and from two other estimates obtained using an independent approach from upscaling a global data set of site-level field measurements [38] by reference [31]. The whisker bars indicate the interquartile range and the full range. (b) Regional estimates of the ratio between soil heterotrophic respiration and NPP for each region with mean and median values of distributions calculated using a Monte-Carlo analysis (see ‘Methods’ section).

Among individual sources of carbon to the atmosphere that are not from SHR, we found that the largest flux is the outgassing by rivers, lakes and estuaries [26,27] ranging from 0.8 to 2.3 PgC yr^−1^. This flux is followed by carbon emissions from fires (1.6 PgC yr^−1^), the consumption of harvested crop products (1.5 PgC yr^−1^), land-use-change emissions (1.0–1.2 PgC yr^−1^), emissions from grazing (1.0 PgC yr^−1^), biogenic reduced-carbon emissions such as methane and volatile biogenic compounds (0.8 PgC yr^−1^) and the decay and burning of wood products (0.7 PgC yr^−1^). Altogether, these fluxes represent a globally large source of carbon to the atmosphere of 8.3 ± 0.9 PgC yr^−1^. From these data combined with NPP (and our bottom-up NEE estimates), we infer a global median value of SHR equal to 39 PgC yr^−1^ with an IQR of 33–46 PgC yr^−1^ and a non-Gaussian uncertainty distribution obtained from a Monte-Carlo analysis across different available data sets and their internal uncertainty (Fig. 2). This new global value of SHR is significantly lower than the estimate of ∼50 PgC yr^−1^ given in previous global assessments [28,29]. To obtain an independent verification of this lower SHR estimation, we upscaled data from 455 site-level measurements of SHR from a global database [30] (see ‘Methods’ section) into regional SHR budgets using two approaches. The first approach is a machine-learning algorithm (Supplementary Text 3) and the other one is an empirical model based on functions of soil moisture and soil temperature fitted to the site data [31] (‘Methods’ section and Supplementary Fig. 7). The two estimates of SHR obtained from site-data upscaling agree well with our value deduced from regional carbon budgets, at both regional and global scales (Fig. 2a), which leads to high confidence that SHR amounts to ∼40 PgC yr^−1^.

## SHR CO_2_ EMISSION IS A SMALLER FRACTION OF NPP THAN IN PREVIOUS ASSESSMENTS

**Figure 3.**
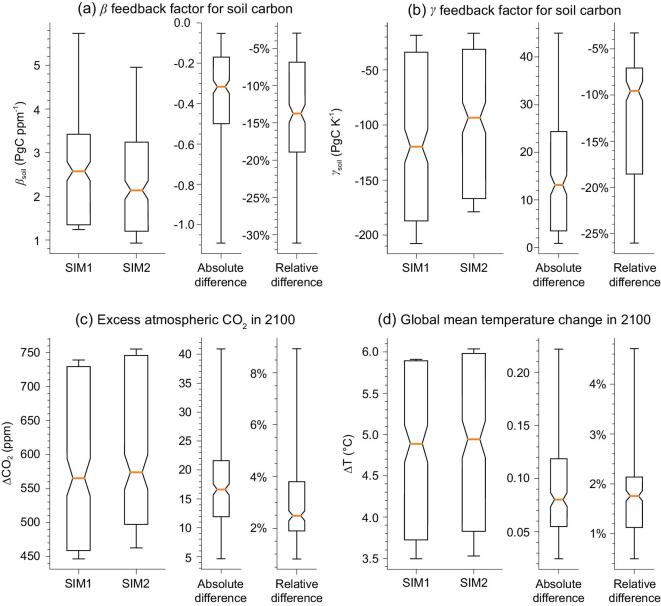
Carbon and climate feedback from soil-carbon simulated with the OSCAR coupled carbon-climate model when emulating seven Earth System Models of the 5th IPCC Assessment Report and following the high fossil-fuel emissions RCP8.5 scenario. In ‘SIM-1’, harvest fluxes, reduced-carbon-compound emissions and riverine export are ignored if not already accounted for in the ESM. In ‘SIM-2’, these lateral fluxes are always included, which altogether have the effect of reducing the flux of litter delivered to the soil and hence soil heterotrophic respiration relative to the net primary production. (a) and (b) β_soil_ is the change in soil-carbon storage per unit change in atmospheric CO_2_ and γ_soil_ is the change in soil-carbon storage in response to a global warming of 1 K. (c) and (d) Atmospheric CO_2_ and global mean temperature by year 2100. The configuration of SIM-1 that corresponds to that of the current-generation Earth System Models leads to under-prediction of the future increase in CO_2_ and temperature. Boxes and whiskers show the interquartile and minimum-to-maximum ranges, from 224 sensitivity simulations of OSCAR (Supplementary Text 4). Both absolute (SIM2 − SIM1) and relative differences (SIM2 / SIM1 − 1) are shown.

The median ratio of SHR to NPP is 0.78 (IQ range from 0.76 to 0.80), which is smaller than the value reported by the third Intergovernmental Panel on Climate Change (IPCC) assessment report [28] of 0.9 and also less than the simulation results from Dynamic Global Vegetation Model (DGVM) carbon-cycle models [5] that give a median of 0.89 and a range of 0.77–0.9. DGVM-based estimates are likely to be overestimated because those models lack a description of the carbon lost to rivers and, in some models, of the components of harvest, which represent carbon not delivered to the soil and thus not available as a substrate for SHR [32]. The ratios of SHR to NPP displayed in Fig. 2b show the lowest values in South Asia (0.38) and East Asia (0.56) where the NPP appropriation by human activities is large, as are fire losses and riverine export. The ratio of SHR to NPP varies between 0.64 and 0.86 in other regions (Fig. 3b). In regions with a large fraction of natural ecosystems like South America and Russia, the values of this ratio are mainly determined by fire losses, reduced-carbon emissions and inland-water outgassing.

Overestimating the ratio of SHR to NPP in global land-carbon-cycle models [5] implies that too much of their NPP is transferred to soils compared to the reality, which gives rise to simulated carbon stocks that are too large and associated turnover times that are too long. However, a positive bias of SHR to NPP in models is not direct evidence that soil-carbon stocks and turnover times in model simulations are inaccurate because of the highly uncertain available soil-carbon observations and compensating errors of model parameterizations. Nevertheless, this structural bias of models should have two consequences for modeling future carbon storage in response to rising CO_2_ and climate change. The first one is that β_soil_ (the change in soil-carbon stock per ppm increase in CO_2_) will be overestimated by models because the overestimated fraction of the future NPP will continue to reach the soil as litterfall, compared to the real world. The second consequence is that models will also overestimate γ_soil_ (the temperature sensitivity of soil carbon, defined by the loss of soil carbon in response to a 1°C global warming) because they over-predict the soil stock exposed to warming. In a coupled carbon-cycle-climate model, overestimating β_soil_ translates into underestimating the amount of CO_2_ that will remain in the atmosphere in the future, whereas overestimating γ_soil_ results in overestimating the amount of CO_2_ released by soil warming in the future.

## IMPLICATIONS FOR THE LAND-CARBON-CYCLE RESPONSES TO RISING CO_2_ AND CLIMATE WARMING

To assess the net effect of these two compensatory effects of overestimating β_soil_ and γ_soil_ on future climate projections, we used the compact coupled carbon-climate model OSCAR [33]. OSCAR was calibrated to reproduce the same pre-industrial regional carbon stocks and NPP, the same sensitivity of NPP to CO_2_ and temperature, and the same sensitivity of soil-carbon decomposition to temperature as seven of the Earth System Models (ESMs) from the 5th IPCC Assessment Report. This calibration ensures that, when run for a future scenario with imposed fossil CO_2_ emissions, OSCAR gives results similar to each original ESM model for future CO_2_ and temperature but at much lower computational costs.

We ran two sensitivity simulations with OSCAR, both forced by fossil CO_2_ emissions from the RCP8.5 pathway [34]. Those emissions lead to an intensive warming and high future CO_2_ concentration, and can be viewed as an extreme test to examine the effect of overestimating β_soil_ and γ_soil_. In the first simulation (SIM-1), SHR was simulated according to each original ESM model (Supplementary Text 4). In the second simulation (SIM-2), we prescribed crop and wood harvest, land use, fires and grazing CO_2_ emissions as a fraction of future NPP in order to reproduce our estimate of present-day SHR-to-NPP ratios for each region (Supplementary Text 4). For the future evolution of riverine-carbon export and outgassing, we constructed an empirical model of this flux based on relationships established by reference [26]. The set-up for SIM-2 is a compromise that avoids a detailed—and uncertain—mechanistic, complex representation of the river export, harvest and grazing. Yet it provides plausible magnitudes for future changes in those fluxes relative to NPP (Supplementary Table 6).

The values of β_soil_ and γ_soil_ calculated by OSCAR from SIM-1 and SIM-2 are shown in Fig. 3 for a combination of seven different ESMs and 32 estimates to account for uncertainty in the SHR-to-NPP ratios for each region. As expected, β_soil_ is 3%–31% lower in SIM-2 than in SIM-1, confirming than SIM-2 systematically reduces soil-carbon storage. Conversely, γ_soil_ is 3%–26% lower (less negative) in SIM-2 than in SIM-1 because there is less carbon in the soil exposed to warming. Although partially cancelling one another out, the reduction of β_soil_ in SIM-2 versus SIM-1 dominates over the reduction of γ_soil_, leading to higher atmospheric CO_2_ in the range of 5–41 ppm in 2100, as shown in Fig. 3c. Similarly, the increase of temperature in 2100 is larger in SIM-2 than in SIM-1, by 0.03–0.22°C (Fig. 3d).

The SIM-2 simulation should be seen as conservative because increasing future wood demand and livestock production in the RCP8.5 storyline [34] should enhance the human appropriation of NPP more than we assumed in the set-up of this simulation. The river-outgassing flux increases significantly in SIM-2, by 61% in 2100 compared to 2000–2009 in response to increased temperature, population and NPP. The development of reservoirs was ignored, which could also lead to both additional outgassing and carbon burial [35]. Presumably, other scenarios such as the RCP2.6, with its vast areas of bio-energy crops and harvested residues, should also significantly affect β_soil_ and γ_soil_.

## CONCLUSION

We conclude that soil carbon is temporally and spatially decoupled from NPP by lateral carbon fluxes from biomass harvest, grazing and carbon export to rivers, as well as by emissions of reduced biogenic carbon compounds. These fluxes are already important today in regions with high human appropriation of NPP and a strong leaching of soil carbon to rivers. The current generation of land-carbon models lack a representation of these lateral fluxes and the underpinning processes, and of their responses to climate change and human pressure. These fluxes are a first-order effect for estimating carbon-climate feedback and we thus recommend incorporating them in the next generation of ESMs and using spatially explicit data sets of harvest and grazing for model evaluation [36]. Likewise, national, regional and global carbon budgets used for the purpose of reporting progress towards targets in climate mitigation all need to account for lateral carbon flows and their consequences for the vertical exchange of carbon between the land biosphere and the atmosphere [37].

## METHODS

### Bottom-up NEE

For each region, NEE is the net flux of carbon exchanged with the overlying atmosphere, excluding fossil-carbon emissions given by Equation (1) in which the total carbon-stock change during the period 2000–2009 (ΔC) is the sum of inventory-based estimates of carbon-storage changes in crop products ${\delta _1}$; in wood products, including products decaying in landfills ${\delta _2}$; in biomass, litter and soil pools ${\delta _3}$; and in inland-water pools corresponding to the carbon burial in sediments ${\delta _4}$. The lithogenic carbon-storage decrease in carbonate rocks from weathering ${\delta _5}$ is not counted in ΔC because it does not contribute to NEE as it does not involve an exchange with the atmosphere, and it was subtracted from the total riverine export of carbon. For ${\delta _1}$, we used data reported by the RECCAP publications in Europe [16] and Australia [15] and assumed zero elsewhere, this storage term being nevertheless very small. ${\delta _2}$ was taken as reported from RECCAP publications [7–14] or calculated by a wood-product-pools bookkeeping model with input being wood products used in each region from reference [39] and decay functions from reference [40]. *δ_3_* was taken from the RECCAP publications [1–18] based on inventories for forest-biomass and data-driven models for soil-carbon changes, for instance models calibrated using soil-carbon inventory data. Those inventories have generally a large uncertainty for soil, litter and dead-wood carbon stocks [41]. For the four tropical regions, changes in soil carbon were ignored and the magnitude of this bias on ΔC is estimated using global models (Supplementary Text 1). Extensive forest-biomass inventories cover northern-hemisphere regions and there are data from research plots in South America and Africa. The primary biomass inventory data are similar to those synthesized over 2000–2007 by reference [42] with some updates to cover the period 2000–2009. In South Asia and Southeast Asia, we used satellite-based estimates of biomass stock changes from Lidar and optical observations [17] updated for this study to a global coverage and averaged over the period 2003–2009 to maximize overlap with the period covered by RECCAP. One-sigma uncertainties of ΔC were taken as reported by the original RECCAP publications (all data given in Supplementary Table 1). Supplementary Fig. 3 presents a comparison of ΔC between RECCAP inventories and the remote-sensing product [17] for each region. Although the remote-sensing estimates of ΔC were only used to fill the gap of missing inventory data in South and Southeast Asia regions, the two independent estimates are within their respective uncertainty for all the other regions, except for Russia, where inventories indicate a larger carbon-storage increase. *δ_4_* is the burial of carbon in freshwater sediments in lakes and reservoirs, reported in the original RECCAP publications only for North America (20 TgC yr^−1^), Europe (41 TgC yr^−1^) and Russia (20 TgC yr^−1^) and here completed by data from reference [43] for other regions.

The bottom-up estimate of NEE was calculated using Equation (1), which requires lateral fluxes from trade and river exports of carbon to oceans, obtained as explained below.

*F_trade_* is the net lateral flux of crop and wood products related to trade across the boundaries of each region, calculated as the sum of the export and import fluxes of crop and wood products. By convention, this flux is positive if a region is a net exporter. For crop products, we considered all products, directly and indirectly, thus covering a broader spectrum of processed crop products than appears in the FAOSTAT database. The carbon in crops is estimated based on FAO crop-production statistics with standard conversion factors to adjust for water and then carbon content [18]. The lateral flux of wood products is calculated in a similar manner, based on reference [18] for different forestry products. For roundwood (FAO code 1861), FAOSTAT data [44] were used and, for the products processed from roundwood and potentially entering international trade, the GTAP-MRIO data [45] were used. This approach considers all products containing roundwood, directly and indirectly, and covers a broader spectrum of processed wood products than appears in the FAO database.

*F_rivers_* is the net export of biogenic carbon by inland waters to the ocean, including dissolved organic carbon (DOC), particulate organic carbon (POC), and dissolved inorganic carbon (DIC). This biogenic carbon export is in fact from atmospheric origin as it was fixed by NPP (predominately terrestrial). The border between inland waters and the ocean is the end of estuaries. The mask of the RECCAP regions is such that there is no river carbon flowing from one region into another, so that imports/export between regions can be ignored. *F_rivers_* was calculated specifically for this study using DOC, POC and DIC at 0.5-degree resolution aggregated into each region, based on the GLOBALNEWS model [19] and data from reference [20]. Following Resplandy *et al.* [46], who recently re-estimated fluvial exports of DOC and POC and showed that these fluxes were underestimated by the GLOBALNEWS model, we used their estimates to rescale spatial explicit estimates from GLOBALNEWS. Only a fraction of DIC transported by rivers is biogenic, the rest being from the lithosphere. The fraction of river DIC from the lithosphere varies in each region because the weathering of carbonate minerals consumes 1/2 mole of atmospheric carbon per mole of DIC transported by rivers, whereas the weathering of silicate minerals consumes 1 mole of atmospheric carbon per mole of DIC transported by rivers. The biogenic and lithogenic proportions of river DIC exports were calculated for this study based on reference [20] and the lithogenic component was subtracted from *F_rivers_*.

To determine SHR from bottom-up estimates of NEE, we used a mass balance equation, whose terms are detailed below:

(2) \begin{eqnarray*} {\rm {SHR}}\ &&= \ {\rm {NEE}} - {\rm {NPP}}\ - {F_{{\mathit {crop}}\ {\mathit {products}}}}\nonumber\\ -\, {F_{{\mathit {wood\ products}}}} - {F_{{\mathit {grazing}}}}\ - {F_{{\mathit{fires}}}}\nonumber\\ -\, {F_{{\mathit{LUC}}}} - {F_{{\mathit{reduced}}}}\ - {F_{{\mathit{outgas}}\ {\mathit {rivers}} + \ {\mathit {lakes}}}}\nonumber \\ -\, {F_{{\mathit{outgas\ estuaries}}}}. \end{eqnarray*}

Uncertainties of SHR were obtained by error propagation from uncertainties of each term from Equation (2), which are independent of each other (see below). A Monte-Carlo approach was used to randomly sample different empirical estimates of component fluxes when available, assuming that each estimate has an equal probability, and then sampling the internal Gaussian uncertainty of each estimate. Different estimates reported in Supplementary Table 1 include four estimates of NPP, two estimates of *F_fires_*, two estimates of *F_LUC_* and two estimates of ${F_{{\mathit{outgas}}\ {\mathit {rivers}}\ + \ {\mathit{lakes}}}}$. Other fluxes have a single estimate and only their internal uncertainty was considered. It is important to note that the error in SHR is positively correlated with the error in NPP and other fluxes, so that the uncertainty of the SHR-to-NPP ratio is lower than that of each flux separately. This is accounted for by the Monte-Carlo approach used to calculate this ratio and shown theoretically (Supplementary Text 5).

NPP was estimated using four different empirical products averaged over each region: the MODIS product [22,47] evaluated against site data [48] reporting an accuracy of ≈20%, the GIMMS product based on AVHRR and MODIS satellite-vegetation-absorbed radiation fraction [24], the BETHY/DLR product based on SPOT-VEGETATION satellite albedo and LAI data [49] and the CARDAMOM carbon-cycle data-assimilation product [25]. Those four NPP products are based on different methods and different sensors. GIMMS and MODIS-NPP use a calibrated light-use-efficiency model while BETHY assimilates vegetation indices in a Soil-Vegetation-Atmosphere model. CARDAMOM does not use satellite observations of NPP. Supplementary Fig. 4 shows that the four largely independent NPP products give consistent estimates in each region, following the Global Climate Observing System definition of consistency [50]: ‘when the independent measurements agree to within their individual uncertainties.’ The global NPP from the four empirical products is also consistent with empirical estimates compiled by references [51] and [52]. We performed a Monte-Carlo analysis by sampling the four different NPP products and assuming for each product a Gaussian uncertainty [48] derived from MODIS-NPP because MODIS-NPP is the only product with a formal uncertainty estimation [48] (relative std. dev. ≈20%). This gives a global median NPP of –50 PgC yr^−1^ with an IQR of –57 to –44 PgC yr^−1^ for the area of all the RECCAP regions (Supplementary Table 1).

${F_{{\mathit{crop\ products}}}}$ is the carbon emission to the atmosphere from the consumption of crop products, calculated as the sum of domestically harvested products minus net export minus storage (*δ*_1_) in each region [18] from FAO data. Crop products are consumed by animals and humans and no distinction was made between these two groups. Digestion of crop products by ruminants emits CH_4_-carbon counted in ${F_{{\mathit{crop\ products}}}}\ $and hence not in ${F_{{\mathit{reduced}}}}$ to avoid double counting. The fraction of consumed products channeled to sewage systems and lost to rivers instead of being emitted to the atmosphere was ignored, as this flux is globally small [21] (100 TgC yr^−1^). The 1-sigma uncertainty of ${F_{{\mathit{crop\ products}}}}{\rm{\ }}$could not be formally established in the absence of global trade-statistics data independently from FAO and was set to 20%.

${F_{{\mathit{wood\ products}}}}\ $ is the carbon emission from the decay and burning of wood products. The term ${F_{{\mathit{wood\ products\ decay}}}}\ $was calculated using a bookkeeping model forced by inputs from the domestic harvest of non-fuelwood [39] minus net export plus imports, to simulate pool changes and losses using decay functions from reference [40]. The small fraction of product waste going to sewage waters and rivers was ignored. The term ${F_{{\mathit{wood\ products\ decay}}}}\ $was calculated in carbon units, including carbon lost to the atmosphere as CH_4_ in landfills. The uncertainty of this flux was set to 20%. ${F_{{\mathit{wood\ products\ burning}}}}$ was calculated from statistics of fuelwood consumption [44] and carbon-emission factors (including CO_2_, CO and CH_4_) compiled in reference [53] and updated to include the fraction of carbon emitted as black carbon [54]. This flux includes commercial fuelwood, fuelwood gathered locally and burned as a fuel in households and industrial fuelwood. The uncertainty of this flux was estimated by accounting for uncertain fuel-consumption and emission factors [53].

${F_{{\mathit{grazing}}}}$ is the carbon emission from the digestion of herbage grazed by animals and the decomposition of manure, here only from grass digestion, because manure from crop-products digestion is already included in ${F_{{\mathit{crop\ products}}}}$. The grass requirement by animals was derived from a grass-biomass-use data set [55] for the year 2000. The evolution of grazed biomass during the period 2001–2009 was calculated based on changes in the total metabolizable energy (ME) of ruminants [56,57]. Actual grass intake was modeled by the ORCHIDEE-GM global model of pasture ecosystems [56] constrained by data from reference [55] including grazing and cut-and-carry forage intake. ${F_{{\mathit{grazing}}}}$ includes CH_4_-carbon emissions from manure and enteric fermentation from reference [58], CO_2_ respiration during grazing and emissions from the use of milk and meat. Animal and manure products are assumed to decay in 1 year. The uncertainty of ${F_{{\mathit{grazing}}}}\ $mainly comes from the grass-biomass-use data [55], obtained from an IPCC tier 3 digestion-metabolism model [59,60] and livestock-population statistics, diet composition and feed quality from databases and surveys. The evolution of the grass-biomass use was estimated based on livestock-population and IPCC tier 2 methods for ME. Uncertainty associated with livestock populations should be <20% and uncertainty of digestibility, a key parameter describing feed quality, is also <20%. Because the uncertainty of the digestion-metabolism model was not estimated, we used the upper-bound uncertainty of the two above input data sources to assess a relative uncertainty of ${F_{{\mathit{grazing}}}}$ of 20%.

${F_{{\mathit{fires}}}}$ is the carbon emission from fires including CO_2_, CO, CH_4_ and black carbon, separated into emissions from crop-residues burning and emissions from other fire types. The residues-burning occurs though small-scale fires that are underestimated by global burned-area products. Further, some residues are burned out of the field and their emissions are missed by satellites. *F_fires crop residues_* was calculated from fuel-consumption and carbon-emission factors [53] updated to include black carbon [54]. Its uncertainty was estimated using a Monte-Carlo method accounting for uncertainties of fuel-consumption and emission factors [53]. Emissions from other fires ${F_{{\mathit{fires\ others}}}}$ were estimated using two different satellite-based data sets: GFED4 (www.globalfiredata.org) based on burned areas [61] and GFAS based on fire radiative power [62]. GFED4 is an update of the GFED3 product [63] with updated burned area [61] completed by a data set of small fires [64]. In tropical regions, deforestation causes fires and those emissions, being already included in ${F_{{\mathit{LUC}}}}$, were subtracted from ${F_{{\mathit{fires\ others}}}}\ $using reference [63] to minimize double accounting. Because the uncertainty of ${F_{{\mathit{fires\ others}}}}{\rm{\ }}$was not formally established, we diagnosed it from the interannual standard deviation of annual emissions in each region during 2000–2009. We verified that this estimate of uncertainty is consistent with formal uncertainties reported for GFED3 [63]. Supplementary Fig. 8 compares GFED4 and GFAS fire emissions in each region.

${F_{{\mathit{LUC}}}}$ is the net land-use-change carbon flux including gross deforestation, secondary ecosystem regrowth, soil CO_2_ emissions after land-use change and forest-degradation emissions, with the latter estimated only for Africa (Supplementary Table 1). *F_LUC_* can be positive or negative, depending on the region considered. *F_LUC_* results from the imbalance between NPP, SHR and deforestation fires over areas historically affected by land-use change. In the absence of NPP and SHR measurements over those areas, *F_LUC_* was treated as a separate flux component of NEE, so that SHR in Equation (2) is not including legacy heterotrophic respiration after LUC, but only respiration on natural and managed lands. We used two different estimates of *F_LUC_*. The first estimate was based on estimates from regional data provided by the RECCAP publications, except for South Asia and Africa, for which the results from the global bookkeeping model of Houghton *et al.* [65] were used. The uncertainty of *F_LUC_* was taken as reported in those publications. The second estimate of *F_LUC_* is from the ‘Bookkeeping of Land Use Emissions’ (BLUE) model described by reference [66], which tracks changes in soil- and biomass-carbon stocks following land-use conversion and due to management practices (e.g. shifting cultivation) using biome-specific carbon densities and exponential response curves. The LUC-transition maps applied were those from LUH2v2.1h [67] from 1850 to 2017. In BLUE, key uncertainties relate to land-use-change areas and carbon densities. We performed sensitivity simulations using different versions of the model to estimate the uncertainty of *F_LUC_* from the BLUE model. For land-use-change input data, the largest uncertainties for the period 2000–2009 (when FAO data and remote sensing enter the LUH2 land-use forcing) arise from shifting cultivation and the allocation of extensive grazing (‘rangelands’) on natural vegetation types, which are both hard to monitor in satellite imagery and inventories. We added and subtracted the difference of simulations with and without shifting cultivation [66] and used two simulations that respectively clear natural vegetation for rangeland expansion or keep natural vegetation intact under this process, the latter as in reference [68]. For carbon densities, we used the original carbon densities of BLUE and an alternative lower map of carbon densities [66]. The standard deviation of the five sensitivity runs plus the default run for each grid cell was used to quantify uncertainty assessment. BLUE data were extrapolated by the last available year (2012 in reference [66] and 2016 in reference [68]). Supplementary Fig. 9 compares RECCAP and BLUE estimates of *F_LUC_* in each region.

${F_{{\mathit{reduced}}}}$ is the emission of reduced carbon, including biogenic CH_4_, non-methane biogenic volatile organic compounds (BVOC) and biogenic CO, excluding fires to minimize double counting. Biogenic CH_4_ emissions from wetlands, termites, rice-paddy agriculture and the small sink in soils were estimated using atmospheric inversions from reference [69]. CH_4_ emissions from crop and wood products in landfills are already counted in ${F_{{\mathit{crop\ products}}}}$ and ${F_{{\mathit{wood\ products}}}}$ and CH_4_ from animals and manure in ${F_{{\mathit{crop\ products}}}}$. The uncertainty of ${F_{{\mathit{reduced}}}}$ was calculated from the standard deviation of 11 inversions [69]. CH_4_ emission from rice paddies was estimated by assuming a fraction of 18% of the total CH_4_ emission from the agriculture and waste source from inversion results [69]. For biogenic carbon emissions BVOC and CO, we used the CLM-MEGAN2.1 gridded product [70] converted into units of carbon mass. CLM-MEGAN2.1 estimates biogenic emissions of CO and of ∼150 BVOC compounds with the main contributions being from terpenes, isoprene, methanol, ethanol, acetaldehyde, acetone, α-pinene, β-pinene, *t*-β-ocimene, limonene, ethene and propene. The uncertainty of that flux was estimated as a percentage of the total flux for each region following sensitivity tests and multi-model comparisons [71], assuming the same relative uncertainty in percentage for all species. The regional uncertainties are: for North America 21%; Europe 35%; Russia 33%; South Asia, East Asia and Southeast Asia 30%; South America 22%; Africa 35%; and Australia 40%. The global emission relative uncertainty is 22%.

*F_outgas rivers + lakes_* is the outgassing of carbon from lakes and rivers. We used two recent spatially resolved estimates of this flux [26,27] calculated using the GLORICH river pCO_2_ database, but with different data-selection criteria and upscaling techniques. The one of reference [27] was made for the COSCAT groups of watersheds, re-interpolated to the RECCAP regions. *F_outgas rivers + lakes_* from reference [26] produced on a 0.5° × 0.5° global grid does not include lakes (lakes were added from reference [27]). River outgassing from reference [26] amounts to half the value of reference [27] due to lower estimates of average river pCO_2_ for the tropics and Siberia resulting from a more restrictive data-selection process and additional averaging effects from the statistical model applied in reference [26]. For estimating SHR (Equation 2), we assumed that carbon emitted by *F_outgas rivers + lakes_* originates entirely from terrestrial NPP. In reality, a fraction of this flux may originate from autotrophic carbon fixation in inland waters [72] and autotrophic respiration from flooded plants [73]. Uncertainty of *F_outgas rivers + lakes_* was taken from original publications [26,27]. It ranges from 20% to 50% of the mean, depending upon the region.

*F_outgas estuaries_* is the outgassing of CO_2_ from estuaries [74] calculated based on a compilation of local flux measurements and a global segmentation of the coastal zone into MARCATS units, which aggregate COSCAT regions. The estimation also accounts for different estuarine types (small deltas, tidal systems, lagoons, fjords). A fraction of *F_outgas estuaries_* may originate from autotrophic C fixation in tidal wetlands [75], which was ignored. The uncertainty of *F_outgas estuaries_* was set to 50%, based on expert judgment.

### Other SHR estimates from site-level data

Two independent estimates of SHR were obtained to be compared with the one obtained from regional carbon data (Fig. 2). The first estimate of SHR is a gridded product from the extension of the approach of Hashimoto *et al.* [29], which estimates soil respiration (SR) (including live-root respiration) using non-linear functions of climate variables and using a global relationship between annual soil respiration and SHR [29,76]. Here, we use the version of this approach by Konings *et al.* [31], for which the parameters of both the climate–SR and SR–SHR relationships were recalculated using site-level data of SR and SHR from the most recent version of the Soil Respiration Data Base [77] (SRDB) (v20180216). These parameters were fitted to SR data from 1979 sites instead of the original 1638 sites in reference [38]. Similarly, 362 measurement sites of SHR distributed across all biomes were used—a much larger data set than the original 53 measurements in the previous version of the SRDB database used by Hashimoto *et al.* [29].

The second SHR gridded product was produced for this study using site-level data from the same SRDB data set upscaled but with a random forest (RF) machine-learning algorithm (Supplementary Text 3). We used 455 site-year observations in total after data filtering (Supplementary Fig. 5) from the following criteria: (i) removing records without complete temporal, coordinates information and annual SHR information; (ii) excluding observations from manipulation experiments and soda-lime-application experiments, which would underestimate soil-CO_2_ fluxes [78]. The RF algorithm applied here is composed of an ensemble of regression trees from bootstrapped training data [79]. We trained RF models with predictor variables including annual temperature, precipitation, soil moisture and radiation, gross primary productivity (GPP), nitrogen deposition, soil-carbon (depths of 0–30 and 30–100 cm) and soil-nitrogen content (Supplementary Table 4). Missing meteorological measurements at SHR sites are filled by gridded climatological driver data based on their coordinates. The values of the remaining predictor variables are extracted at each site location from gridded driving data (Supplementary Table 4). We evaluated the RF-model performance using Leave One Out cross-validation (Supplementary Fig. 6) giving a correlation (*R*^2^) of 0.57 and a RMSE of 111 gC m^−2^ yr^−1^ between observed and predicted values. Simulations of SHR were performed on a 0.5° grid over the globe using gridded predictors fields and averaged for each RECCAP region. The uncertainty of regional mean values of SHR was obtained by modeling the quantiles of this flux using the ‘QuantregForest’ package [80] in R.

## Supplementary Material

nwaa145_Supplement_File
