# Supplementary material for: Empirical estimates of regional carbon budgets imply reduced global soil heterotrophic respiration
Source: Natl Sci Rev. 2020 Jul 7;8(2):nwaa145. doi: 10.1093/nsr/nwaa145 (PMC8288404; doi:10.1093/nsr/nwaa145)
Supplement: nwaa145_Supplement_File [file nwaa145_supplement_file.docx]

Supplementary Information for

**Empirical estimates of regional carbon budgets imply reduced global soil heterotrophic respiration**

Philippe Ciais^*^, Yitong Yao, Thomas Gasser, Alessandro Baccini, Yilong Wang,

Ronny Lauerwald, Shushi Peng, Ana Bastos, Wei Li, Peter A. Raymond, Josep G. Canadell, Glen P. Peters, Rob J. Andres, Jinfeng Chang, Chao Yue, A. Johannes Dolman,

Vanessa Haverd, Jens Hartmann, Goulven Laruelle, Alexandra G. Konings,

Anthony W. King, Yi Liu, Sebastiaan Luyssaert, Fabienne Maignan, Prabir K. Patra,

Anna Peregon, Pierre Regnier, Julia Pongratz, Benjamin Poulter, Anatoly Shvidenko, Riccardo Valentini, Rong Wang, Grégoire Broquet, Yi Yin, Jakob Zscheischler,

Bertrand Guenet, Daniel S. Goll, Ashley-P. Ballantyne, Hui Yang, Chunjing Qiu, Dan Zhu

* Correspondence to: [philippe.ciais@lsce.ipsl.fr](mailto:philippe.ciais@lsce.ipsl.fr)

**This file includes:**

Supplementary Text 1 to 5

Supplementary Table 1 to 6

Supplementary Figure 1 to 9

References

**Supplementary Text 1. Plausible bias of regional NEE from ignoring soil C change in Tropical regions due to a lack of observations**

There is a large uncertainty on soil C changes. Pan et al. [41] analyzed forest C changes used models for SOC changes in absence of data. SOC changes are nevertheless generally smaller than biomass increments in managed tropical forests [105]. No long term tropical SOC changes inventory is available to our knowledge in intact tropical forests.

We can only get a rough estimate of the SOC change contribution to ∆C from models. We looked at the TRENDY v6 DGVM models for SOC changes in the 4 tropical regions and found a) that some TRENDY models are clearly unrealistic with a ratio of SOC change to total carbon change (∆C) of up to 7. When excluding models being 2-sigma outliers in the ensemble for this ratio, the median ensemble values of SOC change to total C change (∆C) during 2000-2009 (Supplementary Table 2) ranges from 13% in S.E. Asia up to 40% in South Asia, with an average of 27%. This gives a rough indication of the omission error in ∆C in our study due to ignoring (non-measured) SOC changes. This error stays within the range of the uncertainty we derived for regional ∆C.

**Supplementary Text 2. Regions not covered by RECCAP region have a small C flux**

These regions cover Greenland, Antarctica, Middle East countries, Ukraine, New Zealand and Kazakhstan. Greenland and Antarctica are ice covered and do not contribute significant C fluxes with the atmosphere. Except for New Zealand, there no systematic observations of the carbon balance of these territories. Middle East and Kazakhstan are dry areas with low forest cover. Ukraine also has a low forest cover. The table below summarizes partial estimates of the carbon balance (mainly ∆C) for these regions.

**Supplementary Text 3. SHR upscaling from site-level measurements using a machine learning approach**

***Data preparation for upscaled SHR estimation****.*

The 455 site-level SRDB observation data are unevenly distributed in boreal, temperate and tropical zones, with the highest sampling density in temperate areas (Supplementary Fig. 5). This dataset covers seven land cover types in total. We tested different predictors and selected those that altogether explained the largest fraction of variance across sites. Table 4 shows the specific data sources of chosen predictor variables, their spatial resolution and temporal resolution. Soil carbon content and soil nitrogen content are static variables without any inter-annual variability. Other variables listed in Supplementary Table 4 are dynamic and we used yearly values.

***Leave-one-out cross validation (LOOCV)***

As mentioned in the Methods section, we applied LOOCV to evaluate whether the model performs well enough at site-level to enable the spatiotemporal SHR prediction at large scales. In the framework of LOOCV, each sample data is used as a validation and compared with the predicted value by a random forest model that is built by all other training samples. The R^2^ and RMSE between the observed and predicted values are shown in Supplementary Fig. 6.

We forced the trained RF model at 0.5° spatial resolution and yearly time step to generate the annual SHR predictions in spatiotemporal grids from 2000 to 2009. The input climatic variables of annual temperature, precipitation and radiation are from CRUNCEP v6. This dataset is a combination of CRU TS3.2 0.5°×0.5° monthly climatological output and NCEP reanalysis data of 2.5°×2.5° and finer temporal resolution of 6-hourly time intervals in near real time. The annual soil moisture input is based on averaging over the CPC monthly soil moisture dataset, which was derived by a leaky bucket model [100]. The annual GPP data is the average of six GPP ensemble data produced by three machine learning methods and two flux partitioning methods in Jung et al [101]. Atmospheric nitrogen deposition data is from the Multi-scale Synthesis and Terrestrial Model Inter-comparison Project environmental forcing dataset [102]. Soil carbon content information is from Harmonized World Soil Database [103]. Both top-soil (0-30cm) and sub-soil (30-100cm) carbon contents are included. Soil nitrogen content data (depth of 0-100cm) is from Global Gridded Surfaces of Selected Soil Characteristics (IGBP-DIS) dataset [104]. The original resolution of soil carbon content (0.05°×0.05°) and soil nitrogen content (0.083°×0.083°) were resampled to 0.5°×0.5° before using in the SHR upscaling procedure.

***Comparison of the two bottom-up SHR upscaled products used in this study***

In addition to Main text Fig. 2 comparing regional average values of bottom-up SHR between the climate based upscaling with re-parameterized Hashimoto et al. [29] functions by Konings et al. [31] and the random-forest based upscaling approach (this study), spatial patterns of annual median SHR from both products are displayed in Supplementary Fig. 7a-b. Desert areas in Sahara, Arabia and Xinjiang in China have been filtered in the Konings et al. products. The spatial distributions of the first (Q1) and third quantiles (Q3) are shown in Supplementary Fig. 7c-f.

**Supplementary Text 4. OSCAR earth system model**

***Model description***

The compact carbon-climate model OSCAR v2.1 [33] was used to estimate the effect of including/excluding processes that prevent carbon from NPP to be delivered as a substrate of soil heterotrophic respiration. This specific version of OSCAR was calibrated to emulate the land carbon cycle of seven ESMs (Supplementary Table 5) with a regional subdivision that follows RECCAP regions (see main text Fig. 1). For each ESM, OSCAR was tuned to match for each RECCAP region the preindustrial land carbon stocks, the turnover times of carbon in biomass and soils, and the NPP of the CMIP5 control run, with unforced variability (named *piControl*). The regional NPP sensitivities to atmospheric CO_2_ (assumed hyperbolic in OSCAR) and to temperature change (assumed linear) are calibrated on the ESMs’ outputs from three idealized simulations of CMIP5 (namely *1pctCO2*, *esmFdbk1*, *esmFixClim1*). The regional *SHR* sensitivities to the input carbon flux from dead biomass (assumed linear), and to temperature change (assumed exponential, i.e. following a Q_10_ formulation) are also calibrated on those three simulations, for each ESM and for each RECCAP region. The climate response to global radiative forcing is emulated by an impulse response function [95] that is calibrated on the CMIP5 simulation *abrupt4xCO2* of the corresponding ESM. Only two correspondences could not be found: so we took CCSM4 as the climate model of the land carbon model CESM-C1 and NorESM-M as that of the land carbon model NorESM-ME.

***Future scenarios SIM-1 and SIM-2 derived from CMIP5 Earth System Model results***

We determine *β*_soil_ and γ_soil_ (shown in main text and. Figure 3) with two simulations giving the increase in atmospheric CO_2_ and prescribing non-CO_2_ radiative forcing of the RCP8.5 [96]. In the first simulation, the carbon cycle and the climate are fully coupled. In the second one, only the climate is impacted by the increase of atmospheric CO_2_ while the carbon cycle sees a pre-industrial climate. *β*_soil_ and γ_soil_ are then calculated following the methodology developed for C4MIP [6]. To determine the increase in atmospheric CO_2_ and temperature (shown in main text Figs 3c and 3d) we used OSCAR in an emission-driven fashion: emissions of CO_2_ from fossil-fuel burning are prescribed following the RCP8.5 data [96].

Two sets of simulations were made in order to estimate the effect of including or not some component fluxes of NEE assessed in this study, that prevent carbon from NPP to be delivered as a substrate of soil heterotrophic respiration. These components are not included in ESMs. In all cases, those fluxes, excepted LUC emissions, are prescribed as a fraction of NPP that is immediately oxidized and emitted to the atmosphere. In the first set of simulations (SIM-1) only the fluxes that are already included in the CMIP5 version of the considered ESM are prescribed (see Supplementary Table 5). In the second set of simulations (SIM-2), all the fluxes are prescribed in each region according to the results synthesized for this study, whatever the ESM.

To perform the SIM-2 simulations with OSCAR being calibrated for each CMIP5 ESM, we need time series of all the NEE components that are not related to SHR, expressed as a fraction of yearly NPP. To create those time series, we first grouped the fluxes of Supplementary Fig. 3 into six groups (Supplementary Table 6). Second, we considered the RECCAP estimates of each flux in each region from Supplementary Table 1 as reference values of the ratio of each flux over NPP, for the 2000-2010 time period. Third, for each of these groups of fluxes, and for NPP, we estimated changes relative to this reference period at different snapshots, using various proxies. The snapshots used to create the time series of the groups of fluxes listed in Supplementary Table 6 are: 1850, 1900, 1950, 2050 and 2100 (following RCP8.5); except for the *Rivers* group that was back-casted only for 1850. Between these points, we used linear interpolation. We used the multi-model mean from the CMIP5 ESMs as proxy for regional NPP (seven models), emissions from wildfires (four models) and CO_2_ fluxes from land-use change and harvested wood products (five models for the past, three for the future). We used the change in cropland and pasture area from the LUH1 dataset [92] as proxies for the *Harvest* and *Grazing* groups of fluxes.

Projections of relative changes in inland water-atmosphere CO_2_ fluxes from 1850, 2000, 2050 and 2100 were specifically made for this study using the spatially-explicit empirical model of ref. [97] with updated values of its main predictor variables: air temperature, terrestrial NPP and population density. The average projected change in NPP and air temperature were derived from the gridded historical data and future projections corresponding to the seven ESMs discussed in this study. Projected changes in population density were obtained from the medium fecundity scenario of the population growth estimated by the UN [98]. The simulated relative changes in CO_2_ evasion were then applied to the present-day estimates of ref. [27] used in this study. For the projections of soil carbon exports to inland waters, it was assumed that the increase in inland water-atmosphere fluxes with time reflects mainly the increased release of non-refractory carbon from soils. Fluvial C exports to the coasts and C burial in sediments are only poorly constrained for 2030 and 2050 [19] and likely reflect more the behavior of refractory soil carbon which is less relevant in the context of this study. In the absence of better constraints, these fluxes were thus assumed constant and, although highly uncertain, the estimated soil carbon change should thus be regarded as conservative. The difficulty in predicting changes in fluvial carbon exports in the coming century is supported by a recent study [99] on the Amazon basin. Finally, we assumed no change in CH_4_ and VOC fluxes in the future, given uncertainties in the sign of these changes. The proxies used for setting changes in the groups of fluxes in the different snapshots, are summarized in Table 3.

Finally, we repeat the above simulation setup for each of the four central estimates of NPP (MODIS, BETHY, GIMMS, CARDAMOM), each of the two central estimates of *F_fires_* (GFED, GFAS), each of the two central estimates of *F_LUC_* (RECCAP, BLUE) and each of the two central estimates of *F_rivers_*. This leads to a total 32 different values of SHR to NPP ratios combined to seven different ESM parameterizations. The final 224 cases are presented in main text Fig. 3.

**Supplementary Text 5. Including error correlations between SHR and NPP for estimating the uncertainty of the ratio of SHR-to-NPP**

The distributions of the ratio SHR to NPP (denoted as *r*) were estimated by error propagation rules. Assuming that both SHR and NPP follow unbiased Gaussian distributions with means *μ* and standard deviation σ so that SHR ~ N(*μ*_SHR_, *σ*_SHR_^2^) and NPP ~ N(*μ*_NPP_, *σ*_NPP_^2^), the mean of *r* for a region *i* is:

$\underline{r_{i}}={\mu_{SHR/NPP}}_{i}=\frac{{\mu_{SHR}}_{i}}{\left| {\mu_{NPP}}_{i} \right|}=-\frac{{\mu_{SHR}}_{i}}{{\mu_{NPP}}_{i}}$

And the first-order approximation for the error of *r* is:

$$\varepsilon_{r_{i}}=\frac{\partial r_{i}}{\partial{(SHR)}_{i}}\varepsilon_{{SHR}_{i}}+\frac{\partial r_{i}}{\partial{(NPP)}_{i}}{\varepsilon_{NPP}}_{i}=-\frac{1}{{\mu_{NPP}}_{i}}{\varepsilon_{SHR}}_{i}+\frac{{\mu_{SHR}}_{i}}{{\mu_{NPP}^{2}}_{i}}{\varepsilon_{NPP}}_{i}$$

The standard deviation of the ratio error is:

$\sigma_{r_{i}}=\sqrt{\left( \frac{1}{{\mu_{NPP}}_{i}} \right)^{2}{{\sigma_{SHR}}_{i}}^{2}+\left( \frac{{\mu_{SHR}}_{i}}{{{\mu_{NPP}}_{i}}^{2}} \right)^{2}{{\sigma_{NPP}}_{i}}^{2}-2\left( \frac{1}{{\mu_{NPP}}_{i}}\cdot\frac{{\mu_{SHR}}_{i}}{{{\mu_{NPP}}_{i}}^{2}} \right)cov\left( {\sigma_{SHR}}_{i},{\sigma_{NPP}}_{i} \right)}$(1)

where *cov(,)* means the covariance of the two variables within the brackets. Since SHR is calculated based on mass balance from NEE, NPP and other non-SHR sources composing NEE noted $F_{ik}$ for *k* = 1, 8 (Supplementary Eq. 2), the covariance between SHR and NPP can be written as:

$$cov\left( {\sigma_{SHR}}_{i},{\sigma_{NPP}}_{i} \right)=cov\left( {SHR}_{i},{NPP}_{i} \right)$$

$=cov\left( {NEE}_{i}-{NPP}_{i}-\sum_{k=1}^{8} F_{ik}, {NPP}_{i} \right)$ (2)

$=cov\left( {NEE}_{i},{NPP}_{i} \right)-var\left( {NPP}_{i} \right)-\sum_{k=1}^{8} cov\left( F_{ik},{NPP}_{i} \right)$

Assuming *NPP* is independent from *NEE* the other fluxes that compose *NEE*, equation (SOM-6) becomes:

$$cov\left( {\sigma_{SHR}}_{i},{\sigma_{NPP}}_{i} \right)=-var\left( {NPP}_{i} \right)={{\sigma_{NPP}}_{i}}^{2}$$

Substituting equation (SOM-7) into equation (SOM-5), the standard deviation of the ratio error is calculated by:

$\sigma_{r_{i}}=\sqrt{\left( \frac{1}{{\mu_{NPP}}_{i}} \right)^{2}{{\sigma_{SHR}}_{i}}^{2}+\left( \frac{{\mu_{SHR}}_{i}}{{{\mu_{NPP}}_{i}}^{2}} \right)^{2}{{\sigma_{NPP}}_{i}}^{2}+2\left( \frac{1}{{\mu_{NPP}}_{i}}\cdot\frac{{\mu_{SHR}}_{i}}{{{\mu_{NPP}}_{i}}^{2}} \right){{\sigma_{NPP}}_{i}}^{2}}$ (3)

$=\frac{\sqrt{\left( {\mu_{NPP}}_{i} \right)^{2}{{\sigma_{SHR}}_{i}}^{2}+\left( {\mu_{SHR}}_{i} \right)^{2}{{\sigma_{NPP}}_{i}}^{2}+2\left( {\mu_{NPP}}_{i}\cdot{\mu_{SHR}}_{i} \right){{\sigma_{NPP}}_{i}}^{2}}}{{{\mu_{NPP}}_{i}}^{2}}$

We further verified that Monte Carlo simulations with random realizations of each flux and SHR calculated as in the main text by mass balance gives the same error in *r_i_* for each region.

**Supplementary Table 1**. Carbon storage change, lateral fluxes from rivers and trade, NEE, component fluxes from NEE, and SHR in each region in Tg C yr^-1^. All data for the period 2000-2009 except for rivers, lakes and estuaries which are based on data covering an epoch of the last two decades.

| **North America** | | | | | |
| --- | --- | --- | --- | --- | --- |
| (TgC yr^-1^) | | | | Reference | Uncertainty estimation |
| ∆C | -348 | ± | 285 | King et al. [7] ^a^ | ^b^ |
| F_trade_ | 30 | ± | 37 | Peters et al. [18] for wood products and SOCCR report [56] for crop products | 20% |
| F_rivers_ | 124 | ± | 47 | Mayorga et al. [27], Hartmann et al. [20] ^c^ | 50% ^d^ |
| NEE | -487 | ± | 291 | Equation (1) | ^e^ |
| F_crop products_ | 182 | ± | 36 | SOCCR report [56] ^f^ | 20% |
| F_wood products_ | 76 | ± | 7 | SOCCR report [56] for wood products decay, Wang et al. for wood burned in power plant and industry [53] ^g^ | ^h^ |
| F_grazing_ | 119 | ± | 24 | This study (see Methods) | 20% |
| F_fires GFED_ | 88 | ± | 33 | Extended from [81] ^i^ | see Methods |
| F_fires GFAS_ | 78 | ± | 23 | Giuseppe et. al. [82] ^i^ | see Methods |
| F_LUC RECCAP_ | -130 | ± | 60 | SOCCR report [56] ^j^ | ^k^ |
| F_LUC BLUE_ | 67 | ± | 14 | Hansis et al. [66] updated in ref. [83] | see Methods |
| F_reduced_ | 71 | ± | 13 | This study (see Methods) | see Methods |
| F_outgas rivers + lakes RAY_ | 219 | ± | 110 | Raymond et al. [84] | 50% |
| F_outgas rivers + lakes LAU_ | 20 | ± | 10 | Lauerwald et al. [26] | 50% |
| F_outgas estuaries_ | 33 | ± | 16 | Laruelle et al. [74] | 50% |
| NPP_MODIS_ | -7513 | ± | 1503 | Zhao et al. [85] | 20% ^l^ |
| NPP_BETHY_ | -8638 | ± | 1728 | Tum et al. [23] | 20% |
| NPP_GIMMS_ | -6477 | ± | 1295 | Kolby Smith et al. [24] | 20% |
| NPP_CARDAMOM_ | -7579 | ± | 2725 | Bloom et al. [25] | 20% |
| Soil Het. Resp. | 6315 | (5063,7664) | | Equation (2) | Inter-quartile from Monte Carlo (Methods) |

a Changes in territorial C stocks including ecosystem C pool and product pool changes "Atmospheric flow method" in Table 1 of King et al. plus burial in lakes and reservoirs from SOCCR Report

b Mean uncertainty of the AIM, TBM and Inventory estimates in King et al.

c F_rivers_ is the C flux transported from estuaries to ocean, obtained by summing biogenic C transport from rivers into estuaries with estuaries C outgassing from Laruelle et al. For C transport from rivers into estuaries, we used Mayorga et al. for DOC and POC and Hartmann et al. for DIC, the sum of this flux rescaled so that all northern hemisphere regions have a river C transport equal to that of Resplandy et al. To separate biogenic river C transport of DIC from total DIC transport, the lithogenic river DIC transport was estimated from Harmann et al. to be 15 Tg C yr^-1^ in this region

d River C transport to estuaries and estuaries C outgassing are both assumed a relative uncertainty of 50%. The total error on F_rivers_ is calculated by assuming these two terms have independent uncertainties

e The total error on NEE is calculated by assuming each term of Equation (1) have independent uncertainties

f F_crop products_ is estimated from crop production (217 TgC yr^-1^) minus crop trade net export (35 TgC yr^-1^) based on SOCCR Report (p. 88)

g F_wood products_ is the sum of wood products decay in landfills, biomass burned in industry and biomass burned in power plants

h Uncertainty assumed to be 50% for landfills wood products and estimated by Monte Carlo approach by Wang et al. for biomass burned in industry and power plants

i F_fires_ includes open fires and crop residues burning estimated from Wang et al.

j F_LUC RECCAP_ includes agricultural soils C balance and a woody encroachment carbon sink from SOCCR Report (Table ES.I pp. 6). Note that changes in forest land-use are imbedded in the ΔC estimate

k From SOCCR uncertainty reported as a 95% CI divided by two to have 1-sigma estimate, assuming Gaussian error

l Based on site level evaluation. The same relative NPP uncertainty was used for other NPP products

| **Europe** | | | | | |
| --- | --- | --- | --- | --- | --- |
| (TgC yr^-1^) | | | | Reference | Uncertainty estimation |
| ∆C | -316 | ± | 158 | Luyssaert et al. [86] ^a^ | Luyssaert et al. |
| F_trade_ | -97 | ± | 17 | Peters et al. [18] | 20% |
| F_rivers_ | 24 | ± | 26 | Mayorga et al. [27], Hartmann et al. [20] | 50% ^b^ |
| NEE | -232 | ± | 161 | Equation (1) | ^c^ |
| F_crop products_ | 272 | ± | 54 | Peters et al. [18] | 20% |
| F_wood products_ | 72 | ± | 19 | Eggers et al. [87] for wood products decay, Wang et al. for wood burned in power plant and industry [53] | ^d^ |
| F_grazing_ | 122 | ± | 24 | This study (see Methods) | 20% |
| F_fires GFED_ | 4 | ± | 2 | Extended from [81] ^e^ | see Methods |
| F_fires GFAS_ | 4 | ± | 1 | Giuseppe et. al. [82] ^e^ | see Methods |
| F_LUC RECCAP_ | -2 | ± | 1 | Schulze et al. [9] ^f^ | Schulze et al. |
| F_LUC BLUE_ | -67 | ± | 9 | Hansis et al. [66] updated in ref. [83] | see Methods |
| F _reduced_ | 14 | ± | 4 | This study (see Methods) | see Methods |
| F_outgas rivers + lakes RAY_ | 166 | ± | 83 | Raymond et al. [84] | 50% |
| F_outgas rivers + lakes LAU_ | 34 | ± | 17 | Lauerwald et al. [26] | 50% |
| F _outgas estuaries_ | 22 | ± | 11 | Laruelle et al. [74] | 50% |
| NPP_MODIS_ | -3029 | ± | 606 | Zhao et al. [85] | 20% |
| NPP_BETHY_ | -3649 | ± | 730 | Tum et al. [23] | 20% |
| NPP_GIMMS_ | -2361 | ± | 472 | Kolby Smith et al. [24] | 20% |
| NPP_CARDAMOM_ | -2268 | ± | 813 | Bloom et al. [25] | 20% |
| Soil Het. Resp. | 1989 | (1435,2614) | | Equation (2) | Inter-quartile from Monte Carlo (Methods) |

a Luyssaert et al. change in stocks in their Table: 9a to j + 10a,b + 11ab + LUC + burial ; change in forest stocks for period 2000-2007 ; other periods for other land use types

b River C transport to estuaries and estuaries C outgassing are both assumed a relative uncertainty of 50%. The total error on F_rivers_ is calculated by assuming these two terms have independent uncertainties

c The total error on NEE is calculated by assuming each term of Equation (1) have independent uncertainties

d Uncertainty from Eggers et al. for landfills and waste wood products and estimated by Monte Carlo approach by Wang et al. for biomass burned in industry and power plants

e F_fires_ includes open fires and crop residues burning estimated from Wang et al.

f See their Fig. 3 (this flux only for EU countries)

| **Russia** | | | | | |
| --- | --- | --- | --- | --- | --- |
| (TgC yr^-1^) | | | | Reference | Uncertainty estimation |
| ∆C | -563 | ± | 218 | Dolman et al. [10] ^a^ | ^b^ |
| F_trade_ | 38 | ± | 11 | Dolman et al. ^c^ | 20% |
| F_rivers_ | 139 | ± | 45 | Mayorga et al. [27], Hartmann et al. [20] | 50% ^d^ |
| NEE | -725 | ± | 223 | Equation (1) | ^e^ |
| F_crop products_ | 60 | ± | 12 | Dolman et al. ^f^ | 20% |
| F_wood products_ | 43 | ± | 14 | Bookkeeping model (see methods) for wood products decay, Wang et al. for wood burned in power plant and industry ^g^ | ^d^ |
| F_grazing_ | 22 | ± | 4 | This study (see Methods) | 20% |
| F_fires GFED_ | 114 | ± | 75 | Extended from [81] ^h^ | see Methods |
| F_fires GFAS_ | 102 | ± | 31 | Giuseppe et. al. [82] ^h^ | see Methods |
| F_LUC RECCAP_ | -34 | ± | 2 | Schulze et al. [9] ^i^ | Dolman et al. |
| F_LUC BLUE_ | -5 | ± | 15 | Hansis et al. [66] updated in ref. [83] | see Methods |
| F_reduced_ | 28 | ± | 7 | This study (see Methods) | see Methods |
| F_outgas rivers + lakes RAY_ | 134 | ± | 67 | Raymond et al. [84] | 50% |
| F_outgas rivers + lakes LAU_ | 17 | ± | 9 | Lauerwald et al. [26] | 50% |
| F_outgas estuaries_ | 18 | ± | 9 | Laruelle et al. [74] | 50% |
| NPP_MODIS_ | -5109 | ± | 1022 | Zhao et al. [85] | 20% |
| NPP_BETHY_ | -6633 | ± | 1327 | Tum et al. [23] | 20% |
| NPP_GIMMS_ | -4291 | ± | 858 | Kolby Smith et al. [24] | 20% |
| NPP_CARDAMOM_ | -5475 | ± | 1989 | Bloom et al. [25] | 20% |
| Soil Het. Resp. | 4163 | (3180,5314) | | Equation (2) | Inter-quartile from Monte Carlo (Methods) |

a Dolman et al. their Table 5 estimate for year 2009 corrected to period 2007-2009 using same IIASA LEA C accounting system (A. Shvidenko pers. Com.)

b Dolman et al. pp.5335 196 TgC yr^-1^ uncertainty 90% CI plus 120 TgC yr^-1^ additional uncertainty added to account for the fact that only 2007-09 is estimated

c Dolman et al. BG 2012 et al. pp. 5332 for wood products and table 5 for crop products

d River C transport to estuaries and estuaries C outgassing are both assumed a relative uncertainty of 50%. The total error on F_rivers_ is calculated by assuming these two terms have independent uncertainties

e The total error on NEE is calculated by assuming each term of Equation (1) have independent uncertainties

f Dolman et al. BG 2012 Table 2 minus "net export" in Table 5

g Uncertainty of 20% for wood products decay and estimated by Monte Carlo approach by Wang et al. for biomass burned in industry and power plants

h F_fires_ includes open fires and crop residues burning estimated from Wang et al.

i See their Fig. 3

| **East Asia** | | | | | |
| --- | --- | --- | --- | --- | --- |
| (TgC yr^-1^) | | | | Reference | Uncertainty estimation |
| ∆C | -300 | ± | 100 | Piao et al. [13] | Piao et al. |
| F_trade_ | -62 | ± | 10 | Peters et al. [18] | 20% |
| F_rivers_ | 88 | ± | 19 | Mayorga et al. [27], Hartmann et al. [20] | 50% ^a^ |
| NEE | -321 | ± | 102 | Equation (1) | ^b^ |
| F_crop products_ | 375 | ± | 75 | Peters et al. [18] | 20% |
| F_wood products_ | 135 | ± | 64 | Bookkeeping model (see Methods) for wood products decay, Wang et al. for wood burned in power plant and industry | ^c^ |
| F_grazing_ | 118 | ± | 24 | This study (see Methods) | 20% |
| F_fires GFED_ | 20 | ± | 5 | Extended from [81] ^d^ | see Methods |
| F_fires GFAS_ | 19 | ± | 6 | Giuseppe et. al. [82] ^d^ | see Methods |
| F_LUC RECCAP_ | -13 | ± | 29 | Piao et al. [13] | Piao et al. |
| F_LUC BLUE_ | 98 | ± | 48 | Hansis et al. [66] updated in ref. [83] | see Methods |
| F_reduced_ | 25 | ± | 6 | This study (see Methods) | see Methods |
| F_outgas rivers + lakes RAY_ | 164 | ± | 82 | Raymond et al. [84] | 50% |
| F_outgas rivers + lakes LAU_ | 29 | ± | 14 | Lauerwald et al. [26] | 50% |
| F_outgas estuaries_ | 8 | ± | 4 | Laruelle et al. [74] | 50% |
| NPP_MODIS_ | -3017 | ± | 603 | Zhao et al. [85] | 20% |
| NPP_BETHY_ | -3654 | ± | 731 | Tum et al. [23] | 20% |
| NPP_GIMMS_ | -2811 | ± | 562 | Kolby Smith et al. [24] | 20% |
| NPP_CARDAMOM_ | -3059 | ± | 1080 | Bloom et al. [25] | 20% |
| Soil Het. Resp. | 1821 | (1307,2384) | | Equation (2) | Inter-quartile from Monte Carlo (Methods) |

a River C transport to estuaries and estuaries C outgassing are both assumed a relative uncertainty of 50%. The total error on F_rivers_ is calculated by assuming these two terms have independent uncertainties

b The total error on NEE is calculated by assuming each term of Equation (1) have independent uncertainties

c Uncertainty of 20% for wood products decay and estimated by Monte Carlo approach by Wang et al. for biomass burned in industry and power plants

d F_fires_ includes open fires and crop residues burning estimated from Wang et al.

| **South Asia** | | | | | |
| --- | --- | --- | --- | --- | --- |
| (TgC yr^-1^) | | | | Reference | Uncertainty estimation |
| ∆C | -124 | ± | 25 | Baccini et al. [17] | see Methods |
| F_trade_ | -7 | ± | 1 | Peters et al. [18] | 20% |
| F_rivers_ | 151 | ± | 44 | Mayorga et al. [27], Hartmann et al. [20] | 50% ^a^ |
| NEE | -253 | ± | 108 | Equation (1) | ^b^ |
| F_crop products_ | 213 | ± | 43 | Peters et al. [18] | 20% |
| F_wood products_ | 65 | ± | 16 | Bookkeeping model (see methods) for wood products decay, Wang et al. for wood burned in power plant and industry | ^c^ |
| F_grazing_ | 121 | ± | 24 | This study (see Methods) | 20% |
| F_fires GFED_ | 9 | ± | 3 | Extended from [81] ^d^ | see Methods |
| F_fires GFAS_ | 10 | ± | 3 | Giuseppe et. al. [82] ^d^ | see Methods |
| F_LUC RECCAP_ | -14 | ± | 50 | Patra et al. [11] | Patra et al. |
| F_LUC BLUE_ | 77 | ± | 6 | Hansis et al. [66] updated in ref. [83] | see Methods |
| F_reduced_ | 25 | ± | 6 | This study (see Methods) | see Methods |
| F_outgas rivers + lakes RAY_ | 40 | ± | 20 | Raymond et al. [84] | 50% |
| F_outgas rivers + lakes LAU_ | 35 | ± | 18 | Lauerwald et al. [26] | 50% |
| F_outgas estuaries_ | 6 | ± | 3 | Laruelle et al. [74] | 50% |
| NPP_MODIS_ | -1291 | ± | 258 | Zhao et al. [85] | 20% |
| NPP_BETHY_ | -1418 | ± | 284 | Tum et al. [23] | 20% |
| NPP_GIMMS_ | -1586 | ± | 317 | Kolby Smith et al. [24] | 20% |
| NPP_CARDAMOM_ | -1443 | ± | 517 | Bloom et al. [25] | 20% |
| Soil Het. Resp. | 577 | (355,826) | | Equation (2) | Inter-quartile from Monte Carlo (Methods) |

a River C transport to estuaries and estuaries C outgassing are both assumed a relative uncertainty of 50%. The total error on F_rivers_ is calculated by assuming these two terms have independent uncertainties

b The total error on NEE is calculated by assuming each term of Equation (1) have independent uncertainties

c Uncertainty of 20% for wood products decay and estimated by Monte Carlo approach by Wang et al. for biomass burned in industry and power plants

d F_fires_ includes open fires and crop residues burning estimated from Wang et al.

| **South East Asia** | | | | | |
| --- | --- | --- | --- | --- | --- |
| (TgC yr^-1^) | | | | Reference | Uncertainty estimation |
| ∆C | 8 | ± | 76 | Baccini et al. [17] ^a^ | see Methods |
| F_trade_ | 81 | ± | 14 | Peters et al. [18] | 20% |
| F_rivers_ | 109 | ± | 62 | Mayorga et al. [27], Hartmann et al. [20] | 50% ^b^ |
| NEE | -169 | ± | 186 | Equation (1) | ^c^ |
| F_crop products_ | 153 | ± | 31 | Peters et al. [18] | 20% |
| F_wood products_ | 61 | ± | 16 | Bookkeeping model (see Methods) for wood products decay, Wang et al. for wood burned in power plant and industry | ^d^ |
| F_grazing_ | 23 | ± | 5 | This study (see Methods) | 20% |
| F_fires GFED_ | 102 | ± | 49 | Van der Werf et al. ^e^ | see Methods |
| F_fires GFAS_ | 106 | ± | 32 | Di Giuseppe et al. ^e^ | see Methods |
| F_LUC RECCAP_ | 350 | ± | 130 | Cervarich et al [14] | Cervarich et al |
| F_LUC BLUE_ | 234 | ± | 17 | Hansis et al. [66] updated in ref. [83] | see Methods |
| F_reduced_ | 76 | ± | 20 | This study (see Methods) | see Methods |
| F_outgas rivers + lakes RAY_ | 507 | ± | 254 | Raymond et al. [84] | 50% |
| F_outgas rivers + lakes LAU_ | 84 | ± | 42 | Lauerwald et al. [26] | 50% |
| F_outgas estuaries_ | 19 | ± | 10 | Laruelle et al. [74] | 50% |
| NPP_MODIS_ | -3380 | ± | 676 | Zhao et al. [85] | 20% |
| NPP_BETHY_ | -3385 | ± | 677 | Tum et al. [23] | 20% |
| NPP_GIMMS_ | -3726 | ± | 745 | Kolby Smith et al. [24] | 20% |
| NPP_CARDAMOM_ | -4254 | ± | 1385 | Bloom et al. [25] | 20% |
| Soil Het. Resp. | 2338 | (1755,2976) | | Equation (2) | Inter-quartile from Monte Carlo (Methods) |

a Includes also ∆C from burial in lakes and reservoirs from Mendoca et al. [43]

b River C transport to estuaries and estuaries C outgassing are both assumed a relative uncertainty of 50%. The total error on F_rivers_ is calculated by assuming these two terms have independent uncertainties

c The total error on NEE is calculated by assuming each term of Equation (1) have independent uncertainties

d Uncertainty of 20% for wood products decay and estimated by Monte Carlo approach by Wang et al. for biomass burned in industry and power plants

e F_fires_ includes open fires and crop residues burning estimated from Wang et al.

| **South America** | | | | | |
| --- | --- | --- | --- | --- | --- |
| (TgC yr^-1^) | | | | Reference | Uncertainty estimation |
| ∆C | 193 | ± | 282 | Gloor et al. [12] ^a^ | Gloor et al. ^b^ |
| F_trade_ | 53 | ± | 9 | Gloor et al. ^c^ | Gloor et al. |
| F_rivers_ | 219 | ± | 79 | Mayorga et al. [27], Hartmann et al. [20] | 50% ^d^ |
| NEE | -72 | ± | 293 | Equation (1) | ^e^ |
| F_crop products_ | 96 | ± | 19 | Mayorga et al. [27], Hartmann et al. [20] | 20% |
| F_wood products_ | 74 | ± | 15 | Bookkeeping model (see Methods) for wood products decay, Wang et al. for wood burned in power plant and industry | ^f^ |
| F_grazing_ | 252 | ± | 50 | This study (see Methods) | 20% |
| F_fires GFED_ | 159 | ± | 51 | Van der Werf et al. ^g^ | see Methods |
| F_fires GFAS_ | 166 | ± | 50 | Di Giuseppe et al. ^g^ | see Methods |
| F_LUC RECCAP_ | 525 | ± | 130 | Gloor et al. | Patra et al. |
| F_LUC BLUE_ | 291 | ± | 84 | Hansis et al. [66] updated in ref. [83] | see Methods |
| F_reduced_ | 302 | ± | 61 | This study (see Methods) | see Methods |
| F_outgas rivers + lakes RAY_ | 719 | ± | 360 | Raymond et al. [84] | 50% |
| F_outgas rivers + lakes LAU_ | 283 | ± | 142 | Lauerwald et al. [26] | 50% |
| F_outgas estuaries_ | 17 | ± | 9 | Laruelle et al. [74] | 50% |
| NPP_MODIS_ | -13462 | ± | 2692 | Zhao et al. [85] | 20% |
| NPP_BETHY_ | -15097 | ± | 3019 | Tum et al. [23] | 20% |
| NPP_GIMMS_ | -14667 | ± | 2933 | Kolby Smith et al. [24] | 20% |
| NPP_CARDAMOM_ | -13228 | ± | 4462 | Bloom et al. [25] | 20% |
| Soil Het. Resp. | 12321 | (10166,14485) | | Equation (2) | Inter-quartile from Monte Carlo (Methods) |

a Gloor et al. their Table 12 + LUC induced ∆C + burial in lakes and reservoirs ∆C from Mendoca et al.

b Uncertainty in ∆C forest biomass (Gloor et al. Table 12), LUC ∆C, burial ∆C added in quadrature; average of 2000-2004 + 2005-2009 errors

c Gloor et al. their Table 11 for wood and Table 10 for crop products

d River C transport to estuaries and estuaries C outgassing are both assumed a relative uncertainty of 50%. The total error on F_rivers_ is calculated by assuming these two terms have independent uncertainties

e The total error on NEE is calculated by assuming each term of Equation (1) have independent uncertainties

f Uncertainty of 20% for wood products decay and estimated by Monte Carlo approach by Wang et al. for biomass burned in industry and power plants

g F_fires_ includes open fires and crop residues burning estimated from Wang et al.

| **Africa** | | | | | |
| --- | --- | --- | --- | --- | --- |
| (TgC yr^-1^) | | | | Reference | Uncertainty estimation |
| ∆C | 3 | ± | 292 | Valentini et al. [8] ^a^ | Valentini et al. ^b^ |
| F_trade_ | -22 | ± | 5 | Peters et al. [18] | Peters et al. |
| F_rivers_ | 96 | ± | 34 | Mayorga et al. [27], Hartmann et al. [20] | 50% ^c^ |
| NEE | -65 | ± | 294 | Equation (1) | ^d^ |
| F_crop products_ | 182 | ± | 36 | Peters et al. [18] | 20% |
| F_wood products_ | 127 | ± | 13 | Bookkeeping model (see methods) for wood products decay, Wang et al. for wood burned in power plant and industry | ^e^ |
| F_grazing_ | 213 | ± | 43 | This study (see Methods) | 20% |
| F_fires GFED_ | 1040 | ± | 52 | Van der Werf et al. ^f^ | see Methods |
| F_fires GFAS_ | 1022 | ± | 52 | Di Giuseppe et al. ^f^ | see Methods |
| F_LUC RECCAP_ | 510 | ± | 100 | Valentini et al. ^g^ | Valentini et al. |
| F_LUC BLUE_ | 299 | ± | 102 | Hansis et al. [66] updated in ref. [83] | see Methods |
| F_reduced_ | 198 | ± | 62 | This study (see Methods) | see Methods |
| F_outgas rivers + lakes RAY_ | 147 | ± | 74 | Raymond et al. [84] | 50% |
| F_outgas rivers + lakes LAU_ | 138 | ± | 69 | Lauerwald et al. [26] | 50% |
| F_outgas estuaries_ | 15 | ± | 7 | Laruelle et al. [74] | 50% |
| NPP_MODIS_ | -10104 | ± | 2021 | Zhao et al. [85] | 20% |
| NPP_BETHY_ | -11531 | ± | 2306 | Tum et al. [23] | 20% |
| NPP_GIMMS_ | -10546 | ± | 2109 | Kolby Smith et al. [24] | 20% |
| NPP_CARDAMOM_ | -10931 | ± | 3558 | Bloom et al. [25] | 20% |
| Soil Het. Resp. | 8274 | (6656,9959) | | Equation (2) | Inter-quartile from Monte Carlo (Methods) |

a ∆C biomass for intact forest from Pan et al. Science 2011 and land use ∆C from Valentini et al. + burial in lakes and reservoirs ∆C from Mendoca et al.

b Pan et al. ∆C-biomass and litter + LUC ∆C + burial ∆C uncertainties added in quadrature

c River C transport to estuaries and estuaries C outgassing are both assumed a relative uncertainty of 50%. The total error on F_rivers_ is calculated by assuming these two terms have independent uncertainties

d The total error on NEE is calculated by assuming each term of Equation (1) have independent uncertainties

e Uncertainty of 20% for wood products decay and estimated by Monte Carlo approach by Wang et al. for biomass burned in industry and power plants

f F_fires_ includes open fires and crop residues burning estimated from Wang et al.

g From Valentini et al. their Table 11 based on Houghton bookkeeping model + an estimate of degradation carbon loss

| **Australia** | | | | | |
| --- | --- | --- | --- | --- | --- |
| (TgC yr^-1^) | | | | Reference | Uncertainty estimation |
| ∆C | -65 | ± | 36 | Haverd et al. [15] ^a^ | Haverd et al. |
| F_trade_ | 11 | ± | 2 | Haverd et al. | 20% |
| F_rivers_ | 3 | ± | 5 | Mayorga et al. [27], Hartmann et al. [20] | 50% ^b^ |
| NEE | -80 | ± | 36 | Equation (1) | ^c^ |
| F_crop products_ | 9 | ± | 2 | Peters et al. [18] | 20% |
| F_wood products_ | 8 | ± | 3 | Haverd et al. for wood products decay, Wang et al. for wood burned in power plant and industry | ^d^ |
| F_grazing_ | 37 | ± | 7 | This study (see Methods) | 20% |
| F_fires GFED_ | 113 | ± | 35 | Van der Werf et al. ^e^ | see Methods |
| F_fires GFAS_ | 106 | ± | 35 | Di Giuseppe et al. ^e^ | see Methods |
| F_LUC RECCAP_ | 18 | ± | 7 | Haverd et al. | Haverd et al. |
| F_LUC BLUE_ | 18 | ± | 7 | Hansis et al. [66] updated in ref. [83] | see Methods |
| F_reduced_ | 53 | ± | 21 | This study (see Methods) | see Methods |
| F_outgas rivers + lakes RAY_ | 13 | ± | 7 | Raymond et al. [84] | 50% |
| F_outgas rivers + lakes LAU_ | 5 | ± | 3 | Lauerwald et al. [26] | 50% |
| F_outgas estuaries_ | 7 | ± | 4 | Laruelle et al. [74] | 50% |
| NPP_MODIS_ | -1792 | ± | 358 | Zhao et al. [85] | 20% |
| NPP_BETHY_ | -1243 | ± | 249 | Tum et al. [23] | 20% |
| NPP_GIMMS_ | -1868 | ± | 374 | Kolby Smith et al. [24] | 20% |
| NPP_CARDAMOM_ | -1563 | ± | 515 | Bloom et al. [25] | 20% |
| Soil Het. Resp. | 1269 | (954,1602) | | Equation (2) | Inter-quartile from Monte Carlo (methods) |

a Haverd et al. from their change of territorial C stocks (biosphere + wood products)

b River C transport to estuaries and estuaries C outgassing are both assumed a relative uncertainty of 50%. The total error on F_rivers_ is calculated by assuming these two terms have independent uncertainties

c The total error on NEE is calculated by assuming each term of Eq. (1) have independent uncertainties

d Uncertainty from Haverd et al. for wood products decay emissions and estimated by Monte Carlo approach by Wang et al. for biomass burned in industry and power plants

e F_fires_ includes open fires and crop residues burning estimated from Wang et al.

| **Globe** | | | | | |
| --- | --- | --- | --- | --- | --- |
| (TgC yr^-1^) | | | | Reference | Uncertainty estimation |
| ∆C | -1547 | ± | 582 | Sum of regions ^a^ | propagated ^b^ |
| F_trade_ | 24 | ± | 29 | Sum of regions | propagated |
| F_rivers_ | 952 | ± | 135 | Sum of regions | propagated |
| NEE | -2438 | ± | 624 | Sum of regions | propagated |
| F_crop products_ | 1542 | ± | 170 | Sum of regions | propagated |
| F_wood products_ | 663 | ± | 78 | Sum of regions | propagated |
| F_grazing_ | 1026 | ± | 82 | Sum of regions | propagated |
| F_fires GFED_ | 1649 | ± | 125 | Sum of regions | propagated |
| F_fires GFAS_ | 1611 | ± | 94 | Sum of regions | propagated |
| F_LUC RECCAP_ | 1211 | ± | 225 | Sum of regions | propagated |
| F_LUC BLUE_ | 1012 | ± | 500 | Sum of regions | (see Methods) |
| F_reduced_ | 792 | ± | 155 | Sum of regions | propagated |
| F_outgas rivers + lakes RAY_ | 2109 | ± | 479 | Sum of regions | propagated |
| F_outgas rivers + lakes LAU_ | 645 | ± | 166 | Sum of regions | propagated |
| F_outgas estuaries_ | 144 | ± | 72 | Sum of regions | propagated |
| NPP_MODIS_ | -48697 | ± | 9739 | Sum of regions | propagated |
| NPP_BETHY_ | -55248 | ± | 11050 | Sum of regions | propagated |
| NPP_GIMMS_ | -48333 | ± | 9667 | Sum of regions | propagated |
| NPP_CARDAMOM_ | -49845 | ± | 6945 | Sum of regions | propagated |
| Median of four NPP | -50300 | (-57600, -44000) | | Sum of regions | Inter-quartile from Monte Carlo (Methods) |
| Soil Het. Resp. | 39100 | (32700,45500) | | Equation (2) | Inter-quartile from Monte Carlo (Methods) |

a The sum of all regions covers the globe excepted Ukraine, Belarus, Kazakhstan, Middle East, Greenland and New Zealand

b Assuming each region has independent uncertainties in bottom-up flux estimates

**Supplementary** **Table 2**. Median SOC to biomass change ratio during 2000-2009 from TRENDY models.

| TRENDY models | S. America | Africa | South Asia | SE Asia |
| --- | --- | --- | --- | --- |
| Median SOC to biomass change ratio during 2000-2009 * | 0.16 | 0.37 | 0.40 | 0.13 |

- Excluding models in the ensemble that have ratios larger than ensemble 2-sigma

**Supplementary Table 3.** Carbon flux estimates of regions not covered by RECCAP publications.

| **Region / country** | **Flux** | **Reference** |
| --- | --- | --- |
| Ukraine - Cropland abandoned (LUC) | -0.008 | Vuichard *et al.* [88] |
| Ukraine (forest) | -0.012 | Shvidenko *et al.* [89] |
| Kazakhstan - Cropland abandoned (LUC) | -0.01 | Flux of Vuichard *et al.* extrapolated to area abandoned Henebry *et al.* [90] |
| Kazakhstan (forest) | -0.04 | Goodale *et al.* [91] (their Fig. 3) |
| New Zealand | -0.021 to 0 | Tate *et al.* [92] |
| All five countries above + Middle East | -0.36 ± 0.4 | Zscheischler *et al.* [93*,*94] |

Units: Pg C yr^-1^ (negative means land carbon accumulation)

**Supplementary Table 4.** Explanatory variables for random Forest based SHR upscaling used in this study.

| Explanatory variables | Temporal resolution | Spatial resolution | Data sources |
| --- | --- | --- | --- |
| annual temperature | yearly | 0.5°×0.5° | CRUNCEPv6 (<http://dods.extra.cea.fr/store/p529viov/cruncep/> ) |
| annual precipitation | yearly | 0.5°×0.5° |  |
| annual radiation | yearly | 0.5°×0.5° |  |
| annual soil moisture content | yearly | 0.5°×0.5° | CPC soil moisture (<https://www.esrl.noaa.gov/psd/data/gridded/data.cpcsoil.html>) |
| annual nitrogen deposition | yearly | 0.5°×0.5° | NACP MsTMIP (<https://daac.ornl.gov/cgi-bin/dsviewer.pl?ds_id=1220>) |
| gross primary productivity | yearly | 0.5°×0.5° | FLUXCOM (Jung et al., 2017)  (<https://www.bgc-jena.mpg.de/geodb/projects/Data.php>) |
| soil carbon content | static | 0.05°×0.05° | Harmonized world soil database (HWSD) (<https://daac.ornl.gov/SOILS/guides/HWSD.html>)  top-soil (0-30cm)  sub-soil (30-100cm) |
| soil nitrogen content | static | 0.083°×0.083° | (<https://daac.ornl.gov/cgi-bin/dsviewer.pl?ds_id=569>)  0-100cm |
| land cover type | static | 0.5°×0.5° | MODIS land cover data (<http://glcf.umd.edu/data/lc/>) |

**Supplementary Table 5.** This table shows whether the six groups of flux components of NEE not related to SHR assessed in this paper were included (i.e. reported) in CMIP5 simulations by the seven ESMs. "Fires" include only wildfires (*F_fires_*), i.e. not anthropogenic biomass burning; "LUC & wood products" stands for land-use change and harvested wood products (*F_wood products_ + F_LUC_*); "Harvest" corresponds to harvest from croplands only (*F_crop products_*); "Grazing" corresponds to animal-driven harvest from pastures (*F_grazing_*). "non-CO_2_" covers fluxes of CH_4_, CO and VOCs (*F_reduced_*). "Rivers" includes all lateral fluxes linked to continental water streams and lakes (*F_outgas rivers_ + F_outgas lakes_ + F_outgas estuaries_*).

|  | **Lateral fluxes included in CMIP5 simulations** | | | | | |
| --- | --- | --- | --- | --- | --- | --- |
| **Models** | **Fires** | **LUC & wood products** | **Harvest**  **of crop** | **Grazing** | **Non-CO_2_**  **Biogenic C-fluxes** | **Rivers** |
| **BCC-CSM-1.1** | False | False | False | False | False | False |
| **CanESM2** | False | False | False | False | False | False |
| **CESM1-C** | True | True | False | False | False | False |
| **HadGEM2-ES** | False | True | False | False | False | False |
| **IPSL-CM5A-LR** | True | True | True | False | False | False |
| **MPI-ESM-LR** | True | True | False | False | False | False |
| **NorESM1-ME** | True | True | True | False | False | False |

**Supplementary Table 6.** Summary of the proxies used to back-cast and forecast the change in the flux components of NEE not related to SHR, relative to the reference period of 2000-2100. Global values of these relative changes are also shown for some points in time.

|  | **Proxy used to assess relative change** | | | | | | |
| --- | --- | --- | --- | --- | --- | --- | --- |
|  | **NPP** | **Fires** | **LUC & HWP** | **Harvest** | **Grazing** | **CH_4_ fluxes** | **Rivers** |
| **Backcast**  **(1850, 1900, 1950)** | CMIP5  (7 models) | CMIP5  (4 models) | CMIP5  (5 models) | LUH1 data | LUH1 data | constant | This study  (see text) |
| **Reference**  **(2000-2010)** | RECCAP | RECCAP | RECCAP | RECCAP | RECCAP | RECCAP | RECCAP |
| **Forecast**  **(2050, 2100)** | CMIP5  (7 models) | CMIP5  (4 models) | CMIP5  (3 models) | LUH1 data | LUH1 data | constant | This study  (see text) |
|  | **Global values relative to reference period** | | | | | | |
| **1850** | 1.00 | 1.14 | 0.53 | 0.14 | 0.11 | 1.00 | 0.85 |
| **2000-2010** | 1 | 1 | 1 | 1 | 1 | 1 | 1 |
| **2050** | 1.87 | 2.05 | 1.14 | 1.06 | 1.05 | 1.00 | 1.31 |
| **2100** | 1.72 | 1.87 | 0.75 | 1.12 | 1.03 | 1.00 | 1.75 |

**Supplementary Figure 1.** Illustration of the calculation of NEE from C storage change and lateral rivers and trade fluxes in equation (1)


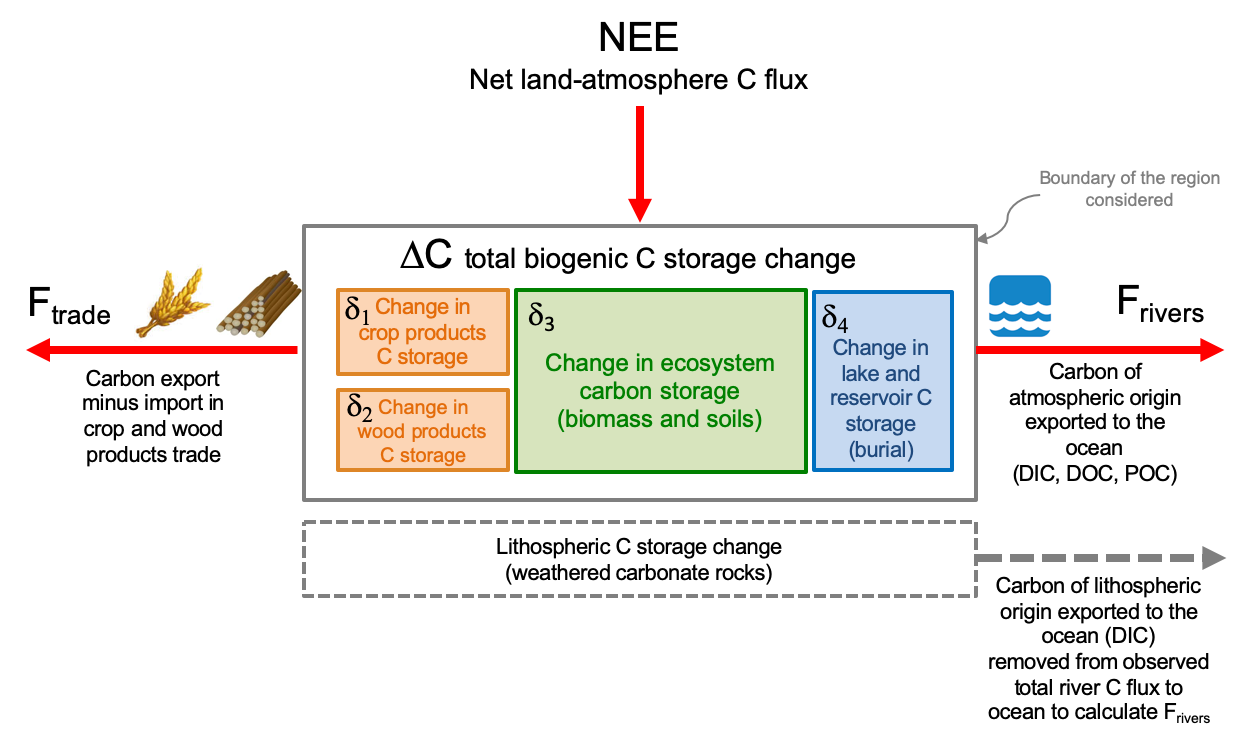


**Supplementary Figure 2.** Comparison of ∆C from inventories based on RECCAP publications (2000-2009) with the satellite-based estimates from ref. [17] covering only biomass C stock changes (2003-2009)


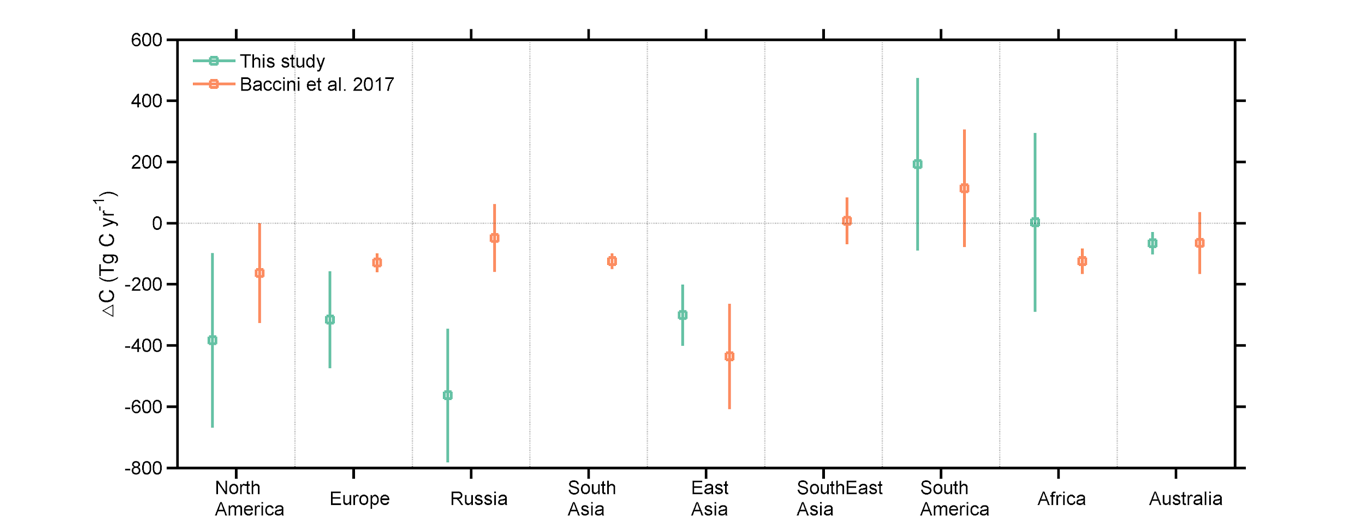

**Supplementary Figure 3.** Decomposition of NEE into component fluxes used to calculate SHR as a residual for each region. Global values of each flux are provided in Pg C yr^-1^ rounded from Supplementary Table 1 to 0.1 Pg C yr^-1^

**Supplementary Figure 4**. Comparison of NPP from four empirical based estimates (2000-2009) for each region (Methods)


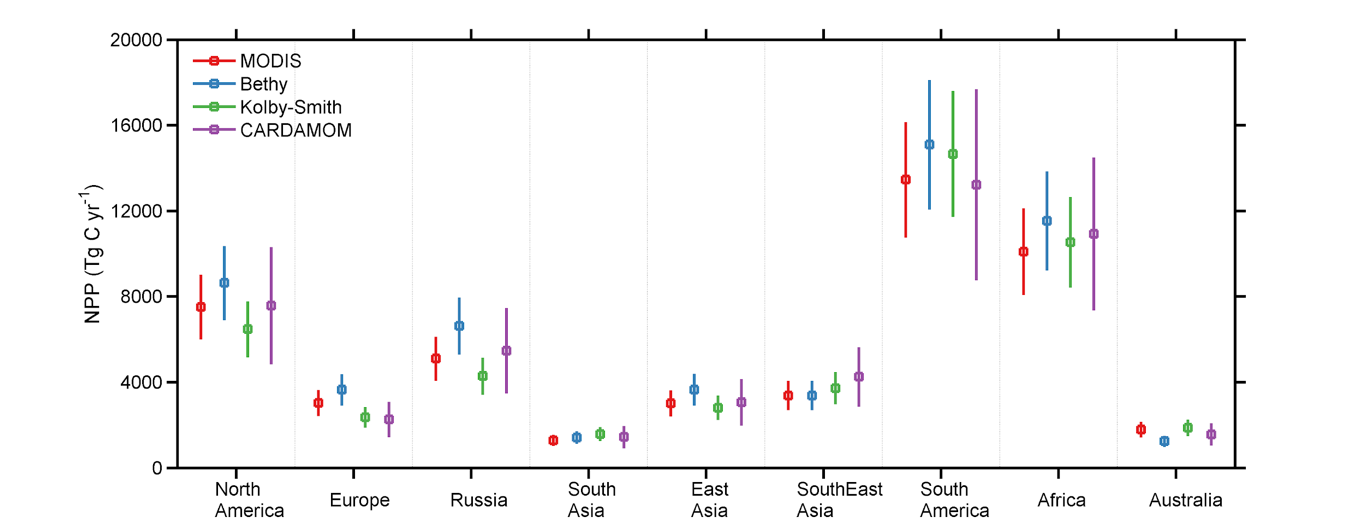


**Supplementary Figure 5** The spatial distribution of SHR observation data used in the Random Forest upscaling (N=455).


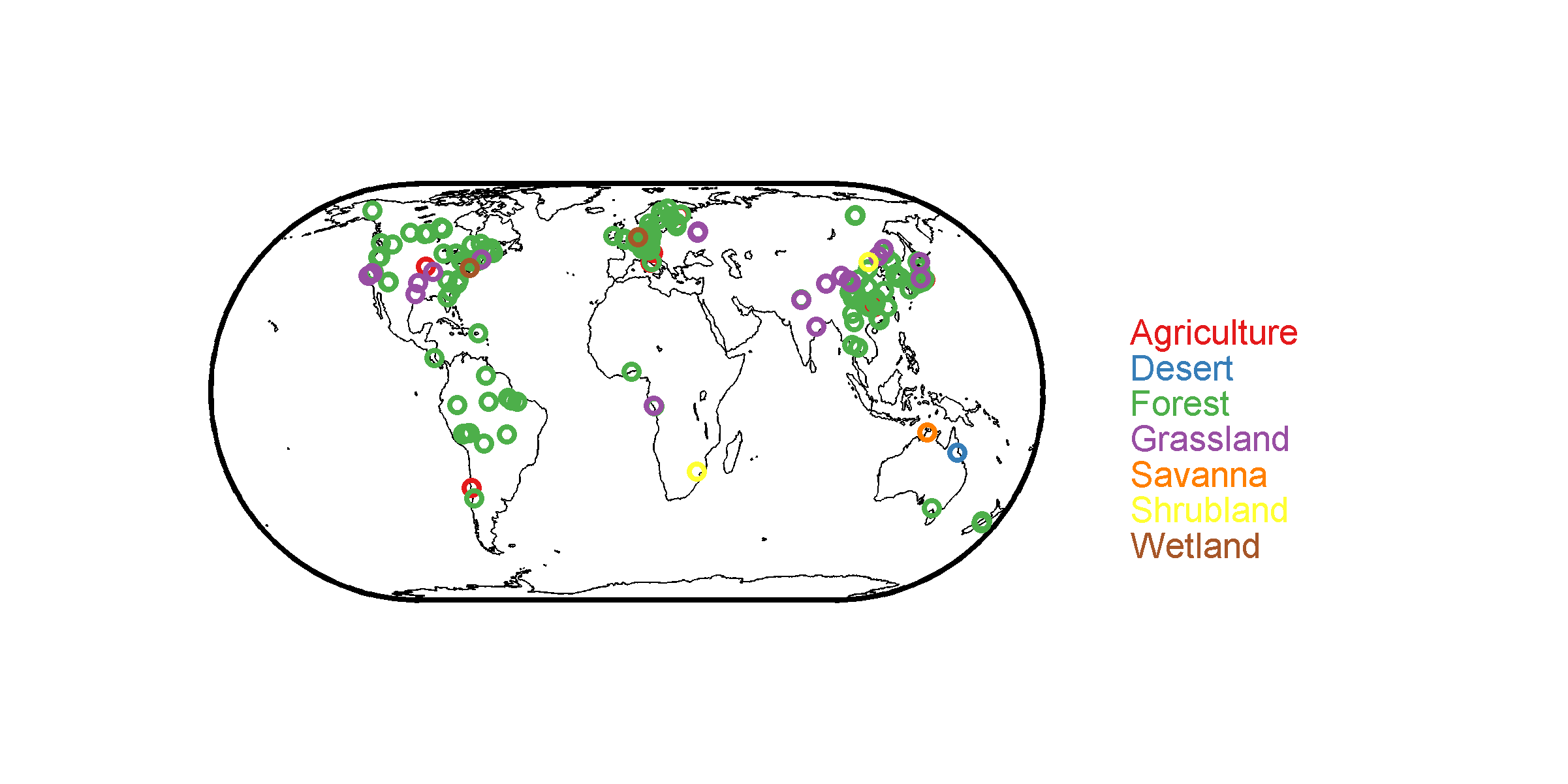


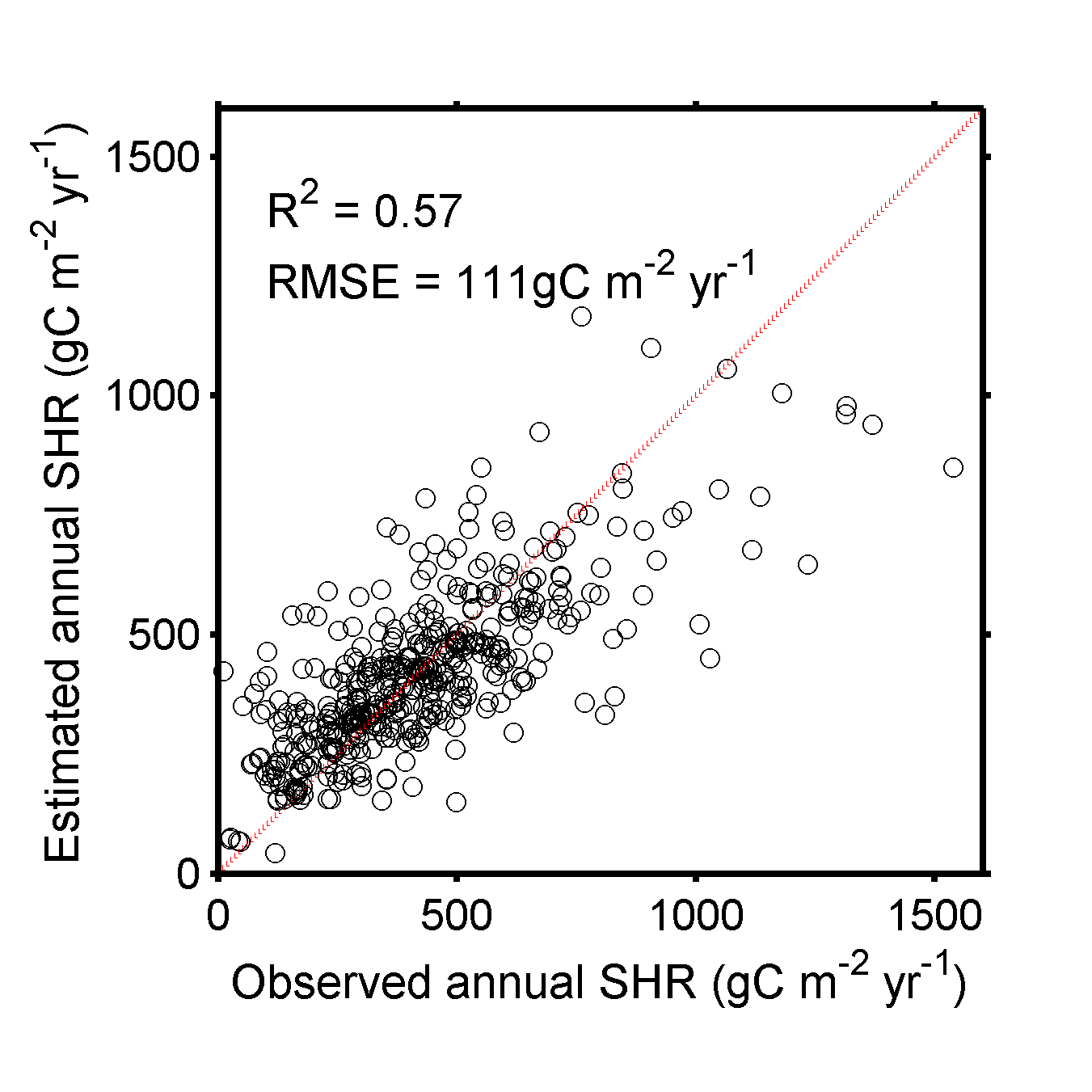
**Supplementary Figure 6** Performance of random forest in estimating site-level annual SHR. The predicted value of each observation is estimated by a random forest model trained by other training samples excluding the validated one.


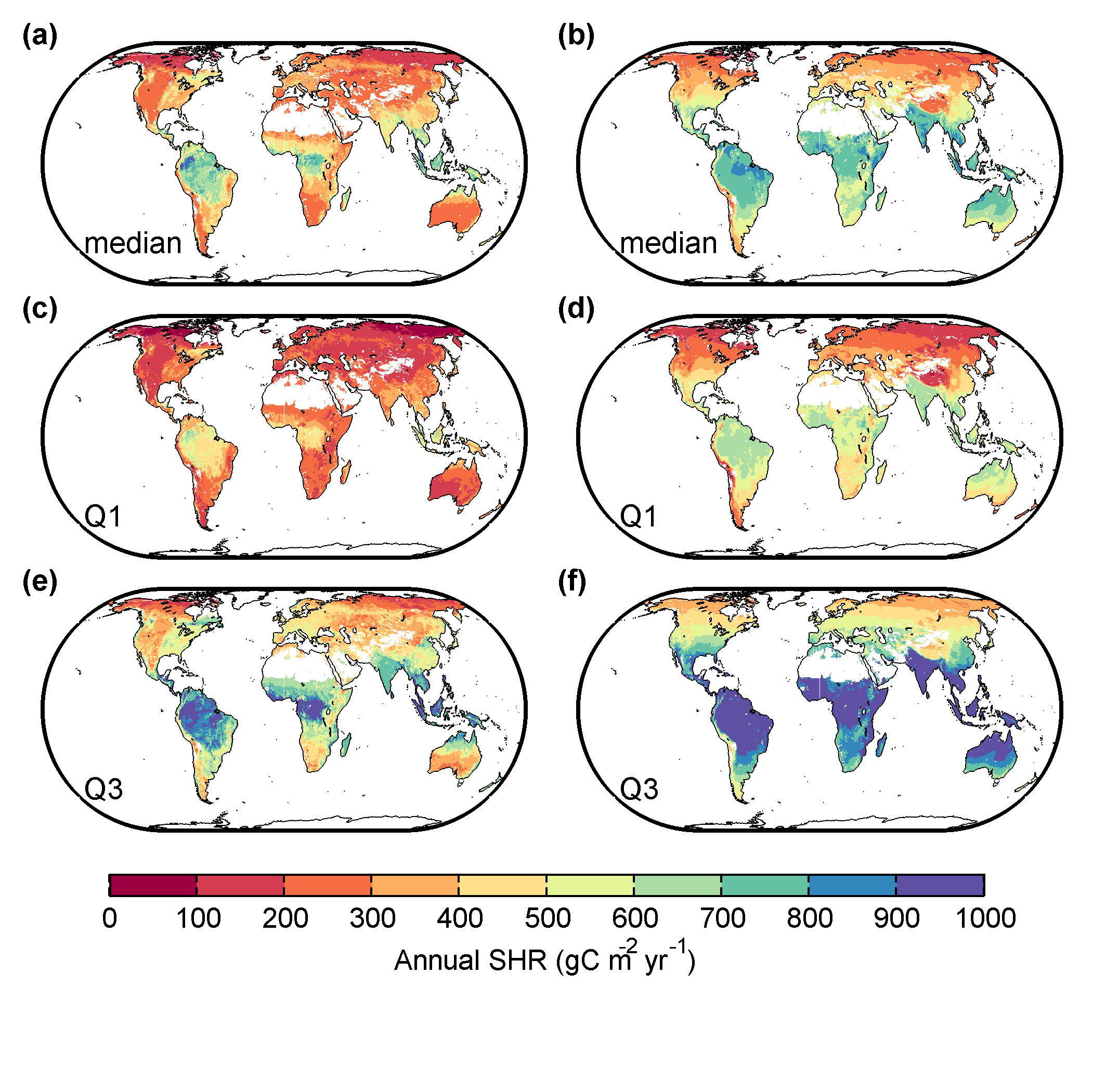
**Supplementary Figure 7.** Spatial distribution of site upscaled SHR from Random Forest in this study **(a,c,e)** and from climate functions as in Konings et al. [31] also used in this study **(b,d,f)**. Median, first quantile, and third quantile are shown.

**Supplementary Figure 8**. Fire C emissions from GFED4 compared with GFAS (Methods)


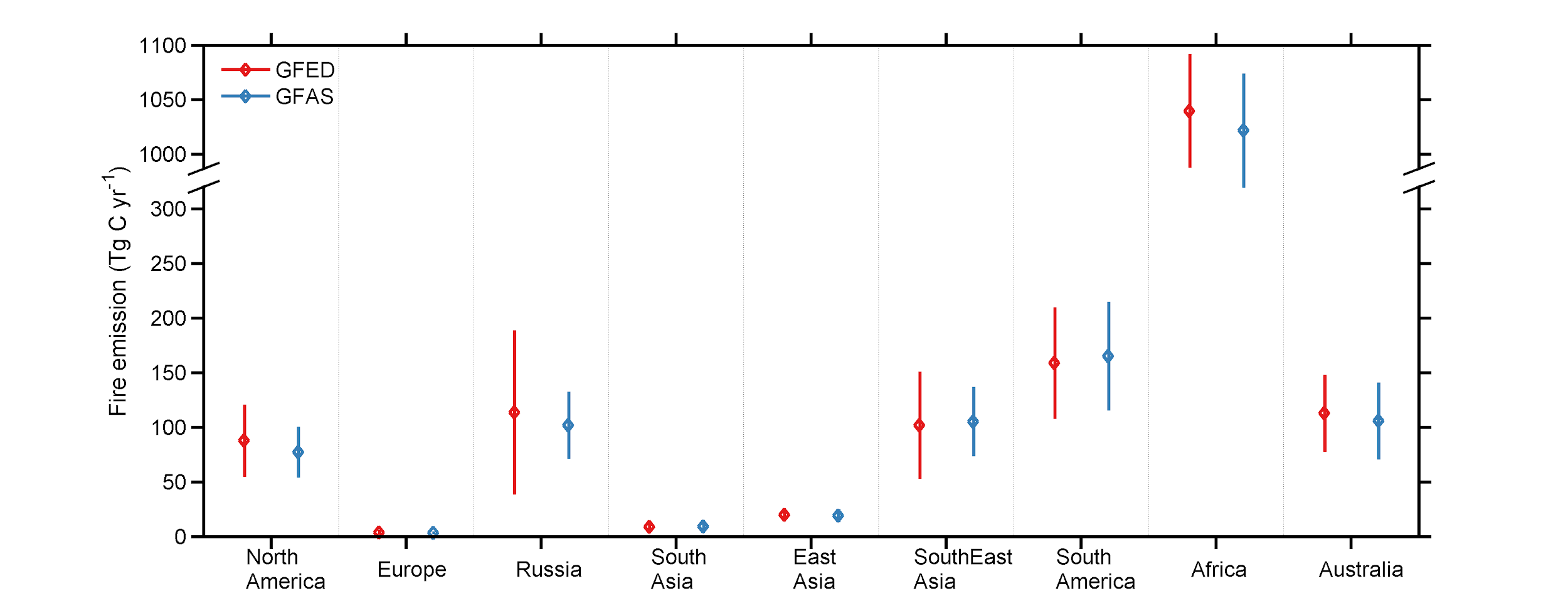


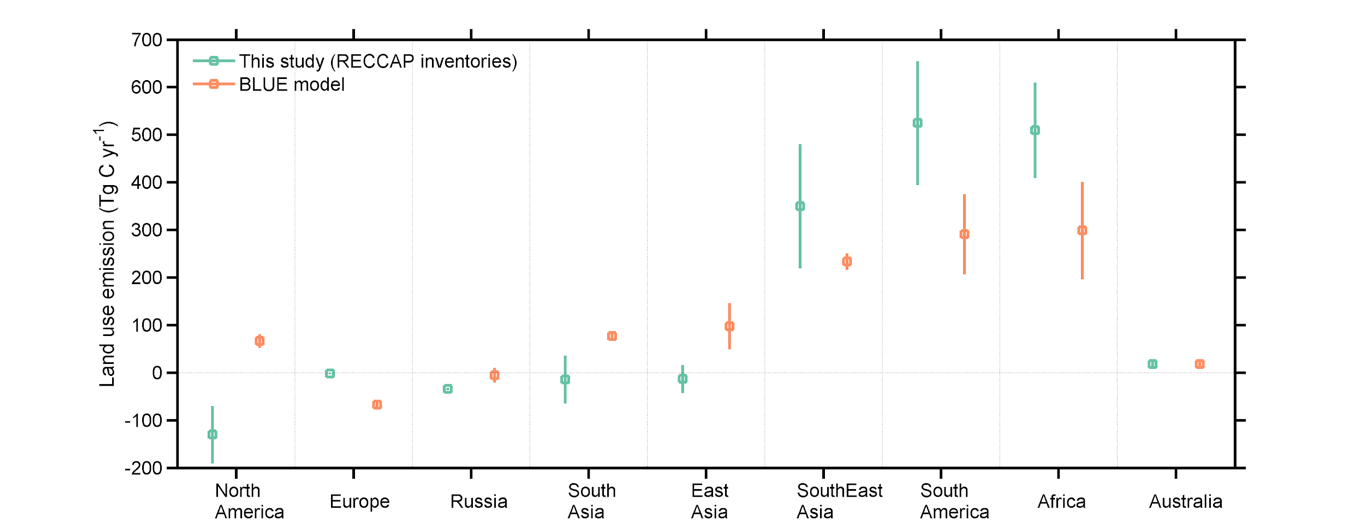
**Supplementary Figure 9**. Net land use change (LUC) emissions from BLUE model compared with RECCAP estimates (2000-2009). Positive values are emissions of carbon to the atmosphere (Methods)

**References**

81. van der Werf GR, Randerson JT and Giglio L *et al*. Global fire emissions and the contribution of deforestation, savanna, forest, agricultural, and peat fires (1997–2009). *Atmos Chem Phys* 2010;**10**:11707–35.

82. Di Giuseppe F, Rémy S and Pappenberger F et al. Using the Fire Weather Index (FWI) to improve the estimation of fire emissions from fire radiative power (FRP) observations. *Atmos Chem Phys* 2018; **18**: 5359–70.

83. Le Quéré C, Andrew RM and Friedlingstein P *et al*. Global Carbon Budget 2018. *Earth Syst Sci Data* 2018;**10**:2141–94.

84. Raymond PA, Hartmann J and Lauerwald R *et al*. Erratum: Global carbon dioxide emissions from inland waters. *Nature* 2014;**507**, DOI: 10.1038/nature13142.

85. Zhao M, Running SW and Nemani RR. Sensitivity of Moderate Resolution Imaging Spectroradiometer (MODIS) terrestrial primary production to the accuracy of meteorological reanalyses. *J Geophys Res* 2006;**111**:G01002.

86. Luyssaert S, Abril G and Andres R et al. The European land and inland water CO2, CO, CH4 and N2O balance between 2001 and 2005. *Biogeosciences* 2012;**9**: 3357–80.

87. Eggers T. *The impacts of manufacturing and utilisation of wood products on the European carbon budget*., 2002.

88. Vuichard N, Ciais P and Belelli L *et al*. Carbon sequestration due to the abandonment of agriculture in the former USSR since 1990. *Global Biogeochem Cycles* 2008;**22**: GB4018.

89. Shvidenko A, Lakyda P and Schepaschenko D *et al*. *Carbon, climate and land-use in Ukraine: forest sector, Korsun-Shevechkivsky, Ukraine [in Ukrainian]*., 2014.

90. Henebry GM. Global change: Carbon in idle croplands. *Nature* 2009;**457**:1089–90.

91. Goodale CL, Apps MJ and Birdsey RA *et al*. Forest carbon sinks in the northern hemisphere. *Ecol Appl* 2002;**12**:891–9.

92. Tate K, Scott N and Parshotam A *et al*. A multi-scale analysis of a terrestrial carbon budget. *Agric Ecosyst Environ* 2000;**82**:229–46.

93. Zscheischler J, Mahecha MD and Avitabile V *et al*. Reviews and syntheses: An empirical spatiotemporal description of the global surface-atmosphere carbon fluxes: Opportunities and data limitations. *Biogeosciences* 2017;**14**:3685–703.

94. Zscheischler J, Mahecha M and Reichstein M *et al*. Towards an purely data driven view on the global carbon cycle and its spatiotemporal variability. *EGU Gen Assem 2015* 2015.

95. Geoffroy O, Saint-Martin D and Olivié DJL *et al*. Transient Climate Response in a Two-Layer Energy-Balance Model. Part I: Analytical Solution and Parameter Calibration Using CMIP5 AOGCM Experiments. *J Clim* 2013;**26**:1841–57.

96. Meinshausen M, Smith SJ and Calvin K *et al*. The RCP greenhouse gas concentrations and their extensions from 1765 to 2300. *Clim Change* 2011;**109**:213–41.

97. Lauerwald R, Laruelle GG and Hartmann J *et al*. Spatial patterns in CO_2_ evasion from the global river network. *Global Biogeochem Cycles* 2015;**29**:534–54.

98. United Nations, Department of Economic and Social Affairs, Population Division (2013).World Population Prospects: The 2012 Revision, DVD Edition.

99. Langerwisch F, Walz A and Rammig A *et al*. Climate change increases riverine carbon outgassing while export to the ocean remains uncertain. *Earth Syst Dyn Discuss* 2015;**6**:1445–97.

100. Huang J, van den Dool HM and Georgarakos KP. Analysis of model-calculated soil moisture over the United States (1931–1993) and applications to long-range temperature forecasts. *J Clim* 1996;**9**:1350–62.

101. Jung M, Reichstein M and Schwalm CR *et al*. Compensatory water effects link yearly global land CO_2_ sink changes to temperature. *Nature* 2017;**541**:516–20.

102. Wei Y, Liu S and Huntzinger DN, *et al.* NACP MsTMIP: Global and North American Driver Data for Multi-Model Intercomparison. ORNL DAAC, Oak Ridge, Tennessee, USA. 2014.

103. FAO, IIASA; ISRIC, ISSCAS. JRC: Harmonized World Soil Database (version 1.2). FAO, Rome, Italy and IIASA, Laxenburg, Austria. 2012.

104. Global Soil Data Task Group. Global Gridded Surfaces of Selected Soil Characteristics (IGBP-DIS). 2000.

105. Marín-Spiotta E and Sharma S. Carbon storage in successional and plantation forest soils: a tropical analysis. *Glob Ecol Biogeogr* 2013;**22**:105–17.
